# Time-Resolved X-ray Absorption Spectroscopy: An MCTDH Quantum Dynamics Protocol

**DOI:** 10.1021/acs.jctc.3c00953

**Published:** 2023-12-15

**Authors:** Francesco Segatta, Daniel Aranda, Flavia Aleotti, Francesco Montorsi, Shaul Mukamel, Marco Garavelli, Fabrizio Santoro, Artur Nenov

**Affiliations:** † Dipartimento di Chimica Industriale “Toso Montanari”, University of Bologna, Viale del Risorgimento, 4, 40136 Bologna, Italy; ‡ ICMol, Universidad de Valencia, c/Catedrático José Beltrán, 2, 46980 Paterna, Spain; § Istituto di Chimica dei Composti Organometallici (ICCOM-CNR), Area della Ricerca del CNR, Via Moruzzi 1, I-56124 Pisa, Italy; ∥ Department of Chemistry and Department of Physics and Astronomy, University of California, Irvine, 92697 California, United States

## Abstract

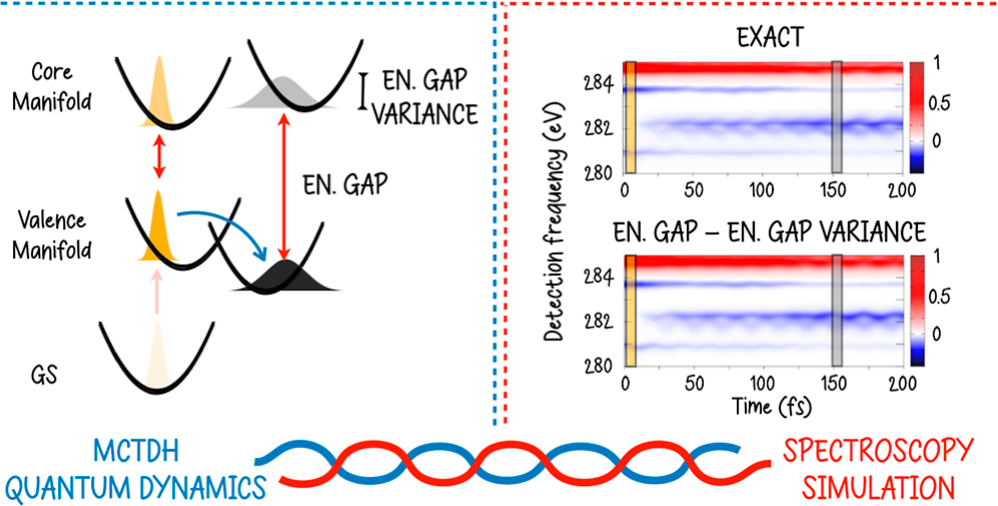

Expressions for linear and nonlinear spectroscopy simulation in the X-ray window in which the time evolution of a photoexcited molecular system is treated via quantum dynamics are derived. By leveraging on the peculiar properties of core-excited/ionized states, first- and third-order response functions are recast in the limit of time-scale separation between the extremely short core-state lifetime and the (comparably longer) electronic-state transfer and nuclear vibrational motion. This work is a natural extension of Segatta et al. (*J. Chem. Theory Comput.***2023,***19,* 2075–2091), in which some of the present authors coupled MCTDH quantum dynamics to spectroscopy simulation at different levels of sophistication. Full quantum dynamics and approximate expressions are compared by simulating X-ray transient absorption spectroscopy at the carbon K-edge in the pyrene molecule.

## 1 Introduction

Monitoring photoinduced events in real time is the primary goal of time-resolved (TR-) spectroscopy. Transient absorption (TA) is one of the most widespread techniques: an ultraviolet (UV)/visible (vis) *pump* pulse excites the system into the manifold of valence-excited states where the photophysics/photochemistry of interest occurs. After a controlled time delay, a *probe* pulse is sent to the sample to monitor the evolution of the molecular system that took place during the delay time. Techniques interrogating the system’s temporal evolution with infrared (IR) or UV/vis light are routinely used to resolve events taking place on the sub-ps time scale,^2–4^ demonstrating that a wealth of complementary information can be obtained by targeting vibrational levels (IR) and valence-excited and -ionized states (UV/vis). Recently, new sources of light, including X-rays in free-electron lasers (XFEL)^5,6^ large facilities and table-top high harmonic generation (HHG)^7,8^ setups, have facilitated access to a new spectral probing window that covers core-excited and -ionized states.^9–15^ By employing X-rays as a probe, promotion of (atom-centered) core electrons into empty molecular orbitals (core excitations) or in the continuum (core ionization) reveals information about the photoinduced dynamics of the system, which is inaccessible by probing other spectral regimes. Such information is *local*, atom-dependent, and sensitive to the chemical environment and/or oxidation state of the core-excited atom.

The simulation of TA signals requires computation of the nonlinear (third-order) response of the system perturbed by the pump and probe laser pulses. The pump pulse creates an out-of-equilibrium wave packet (WP) in the manifold of valence states, whereas the probe resonantly couples the evolved WP to a manifold of higher vibrational and/or electronic excited states. Regardless of the energy window that different types of probe may employ to monitor the photoinduced process of interest, it is understood that the quality of the simulated TA spectra will be strongly affected by the quality of the WP evolution. While cost-effective semiclassical^16^ or mixed quantum-classical^17,18^ approaches have been developed in the last few decades to replace the WP propagation with a swarm of trajectories, the highest level of accuracy is obtained by means of quantum dynamics methods, in which the quantum nature of both electrons and nuclei is preserved. Interestingly, many approximate approaches are not capable of capturing subtle quantum effects such as the creation of electronic coherences along the dynamics, as, e.g., when the WP passes through a conical intersection. Novel spectroscopic techniques in the X-ray domain (such as TRUECARS and ultrafast X-ray diffraction)^19–21^ that leverage on the detection of such coherences to pinpoint passage through a conical intersection require these to be carefully described, making it necessary to employ quantum dynamics WP propagation schemes. Recently, we have developed a simulation protocol for computing the third-order response from multidimensional fully quantum dynamical simulations based on the multiconfigurational time-dependent Hartree (MCTDH) method.^1^ In the present contribution, the approach of ref (1) is further developed to conjugate the accuracy of quantum dynamics simulations with an efficient TA simulation protocol in the X-ray domain.

MCTDH^22–24^ is a numerical protocol for solving the time-dependent Schröedinger equation which is particularly effective when the potential energy surfaces (PESs) on which the nuclei evolve can be approximated by a low-order Taylor expansion in normal mode coordinates. This is often possible in the diabatic picture, in which the molecular system is represented in a basis of states with well-defined (electronic) character. Hamiltonians that describe such simplified PESs are called model vibronic coupling Hamiltonians.^25,26^ The simplest form of the Hamiltonian, known as the linear vibronic coupling (LVC) model, assumes that all PESs share the same normal modes and frequencies and that off-diagonal terms, emerging in the diabatic representation, are linear functions of the coordinates. This facilitates QD simulations with many tens of nuclear degrees of freedom. Quadratic vibronic coupling (QVC) models are also efficiently treated at the MCTDH level, also taking into account different curvatures for the different electronic states, and even Duschinsky mixing between normal modes.^27,28^ MCTDH can treat more general PES functional forms, inevitably requiring reduction of the dimensionality of the problem due to the increased computational cost.

Despite its simplicity, the LVC Hamiltonian naturally captures passage through conical intersections, which is known to facilitate ultrafast internal conversion, as well as charge and energy transfer.^29^ In our implementation, the LVC Hamiltonian is parametrized with a maximum-overlap diabatization protocol,^30,31^ using multiconfigurational wave-function-based electronic structure methods such as the complete and restricted active space self-consistent field theory corrected by second-order perturbation theory, i.e., the CASSCF/CASPT2 and RASSCF/RASPT2 protocols.^32,33^ The response function computation requires evaluation of the overlap between nuclear WP (WPO) evolving on different electronic surfaces during the pump–probe delay time, *t*_2_, as well as during the time that elapses between the probe–matter interaction and the signal detection, termed *t*_3_. This LVC/WPO protocol can be applied to simulate TA signals in the entire spectral window from the visible to the X-ray.

The short-lived nature of core-excited states compared to the time scales of electronic population dynamics and nuclear vibrational periods justifies treating the WP propagation following the interaction with the probe pulse in approximate ways. The short-time approximation (STA) invoked in ref (34) computes static absorption signals on top of a WP dynamics in the valence manifold, thereby completely neglecting the WP evolution projected by the probe pulse in the manifold of higher-lying states. Its validity for X-ray probe pulses has been studied by Freibert et al.^35^ who compared LVC/STA results with the highest LVC/WPO approach. They report that STA accurately reproduces the positions of the transient signals and their broadening, but as expected it lacks a fine vibronic structure, which becomes more visible the longer the lifetime of the core-excited states.

The exact STA response at each delay time *t*_2_ can be obtained either by computing the nuclear probability density in coordinate space^36,37^ at a cost which increases with the number of degrees of freedom, or, alternatively, in the time domain by explicitly propagating the WP during a time interval *t*_3_, while omitting the kinetic operator, at a cost comparable to that of the WPO method.^35^ An approximation often made is to utilize only the vertical energy gap at the centroid, i.e., at the expectation value of the nuclear position of the WP evolving in the manifold of valence states. While this approximation minimizes the overhead to the *t*_2_ WP propagation in the valence manifold, peak broadening and/or asymmetries due to the form of the nuclear probability density are fully neglected. Instead, the stick spectra obtained are dressed with phenomenological broadening, set identical for all valence–core transitions and constant in time.^38^ In the following, we will refer to this level of approximation as the (vertical) energy gap (at centroid) approximation (EGA).

In this contribution, we develop a protocol which improves the EGA by incorporating a time- and transition-dependent analytical signal broadening due to WP *width* in the valence states (accurately captured by the quantum dynamics evolution of the WP) by introducing an energy gap (pseudo)variance expression whose exact form is rigorously obtained by Taylor expanding the WP propagator during the *t*_3_ time interval. We refer to this protocol as the energy gap/(pseudo)variance (at centroid) approximation (EGVA). The performance of this approach is assessed by comparing X-ray TA spectra obtained with the LVC/EGVA and LVC/WPO protocols at the carbon K-edge of pyrene, following the ultrafast (sub-100 fs) S_2_ → S_1_ internal conversion (IC) in UV-excited pyrene.^31,39–43^

## 2 Methods

### 2.1 Quantum Chemistry of Core Excitations

The required information for the simulation of X-ray pump–probe spectra within the LVC model includes state energies and gradients, as well as transition dipole moments (TDMs) between electronic states of different manifolds (see Figure 2a). The RASSCF/RASPT2 parametrization of the LVC model including the seven lowest valence-excited states has been described elsewhere.^31^ The core-excited states were calculated following the protocol documented in ref (44). The high-lying core-excited states were directly obtained at the RASSCF level by putting one core orbital at a time in an orbital subspace (RAS1) and excluding from the configurational space configuration state functions (CFSs) in which it is doubly occupied (by means of the HEXS keyword); moreover, we avoided possible rotations of the core orbital out of the active space (by means of the SUPSYM keyword), which might occur as the RASSCF optimizer will favor their substitution with “higher-lying” inactive valence orbitals (to describe lower-lying valence-excited states), paying the price of freezing the considered core orbital in its SCF shape. Their energy was corrected by applying the multistate version of RASPT2 (with the FROZEN = 0 option).

Specifically, due to the *D*_2*h*_ symmetry of pyrene in its ground-state minimum, one only needs to consider core excitations from the five nonequivalent carbon centers (see Section S1 of the Supporting Information for the pyrene structure and the five carbon centers): for each of them, a 15-state RASSCF/RASPT2 calculation was performed putting the corresponding 1s core orbital in RAS1, resulting in a total of 75 core-excited states. The rationale behind this choice is based on the spectral window we decided to simulate (the pre-edge region between 275 and 285 eV, which is expected to be less congested with respect to the region above the carbon IP energy, which lies at about 290 eV), making the computation of additional higher-lying core-excited states not relevant. The core-excited manifold was computed with *C*_*s*_ symmetry: this allows us to describe the relaxation effect that involves the valence orbitals upon core excitation, with a considerable electron density redistribution on the top of the created core-hole and a consequent stabilization of the core-excited state energies. Solutions that exactly respect the *D*_2*h*_ symmetry would not be capable of describing this orbital localization effect. Moreover, in pyrene, most of the equivalent (isoenergetic) cores lie in distant parts of the molecule, making their overlap negligible and any possible delocalization effect not relevant in this specific case. Within the *C*_*s*_ symmetry, all π and π* orbitals belong to the *a*″ irreducible representation, while core orbitals belong to *a*′. The low-lying valence states of ππ* nature will therefore be of *A*′ symmetry, while the core-to-π* excited states will be of *A*″ symmetry. The lower symmetry makes impracticable computations with the extended active space (i.e., full-π augmented with a set of virtual orbitals) reported in our previous study of the valence manifold (ref (31)); therefore, it was reduced to the (full-π) 16 frontier π and π* orbitals (16 electrons, with maximum four excitations), plus the carbon 1s orbital bearing two additional electrons [with maximum one excitation; the final active space will be labeled as RAS(1,1|4,8|4,8)]. Moreover, the high number of core-excited states and the increased cost of RASPT2 without frozen orbitals make the calculation of RASPT2 gradients (either numerical or analytical) unfeasible; therefore, we relied on RASSCF gradients for these states.

In order to compute TDMs between valence and core states, they all have to be obtained at the same level of theory (active space size and composition); therefore, their calculation was repeated as outlined above, keeping the desired core orbital in RAS1 but including CSFs with doubly occupied core orbitals in the configurational space (i.e., not employing the HEXS keyword).

In passing, we note that a similar procedure can be employed to compute core-ionized states, required for simulating X-ray photoelectron spectroscopy (XPS) and time-resolved XPS (TR-XPS) spectra. In that case, instead of TDMs, one could evaluate Dyson orbital norms as the approximate ionization cross-section. The protocol is detailed in ref (44).

All electronic structure calculations were performed with OpenMolcas^45–47^ using the generally contracted relativistic atomic natural orbital basis set (ANO-RCC^48^ with 4s3p2d and 2s1p contraction for C and H atoms, respectively) and applying Cholesky decomposition in the calculation of two-electron integrals. In all RASPT2 calculations, the imaginary shift was set to 0.2 au and the IP/EA shift to zero.

Quantum dynamical propagation of vibronic WPs was performed with the MCTDH method as implemented in the code Quantics.^49^ We adopted a primitive basis set of Hermite DVR functions and a multiset formulation for the single particle functions (SPFs) which make it easier to project the part of the WP residing on a specific valence state *e* to a specific core-excited state *c*. As for the integrator section, we used a constant mean field approach, a Bulirsch–Stoer extrapolation integrator for SPFs, and a short iterative Lanczos for the multiconfigurational *A* coefficients.^22^

### 2.2 Spectroscopy Simulations

By leveraging the time-scale separation between core-excited state lifetime (from sub-fs to a few fs) and electronic-state transfer/vibrational motion of the nuclei (with hydrogen bonds exhibiting the shortest vibrational period of about 10 fs), we now derive the approximate energy-gap/energy-gap variance expressions for the evaluation of first- and third-order response functions, required for the simulation of linear and TA spectroscopy. The basic derivation steps are reported here; additional details are provided in the Supporting Information.

#### 2.2.1 System Hamiltonian and Wave Functions

**Figure 1:**
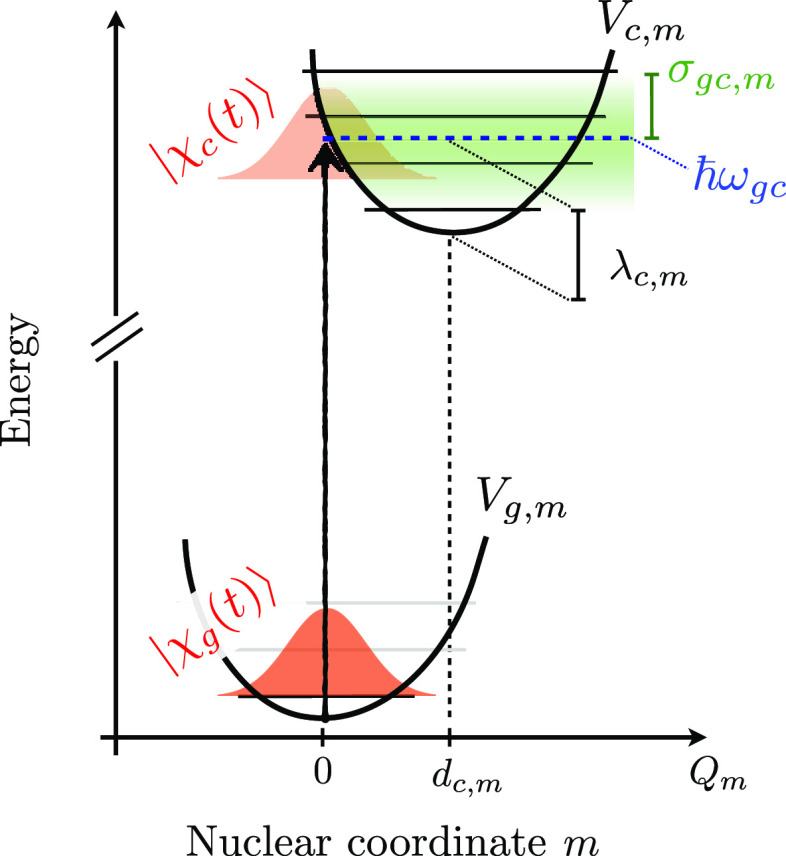
Schematic representation of the potentials *V*_*g*,*m*_ and *V*_*c*,*m*_ along a given mode *m*, the displacement *d*_*c*,*m*_, the vertical energy gap ℏω_*gc*_, the reorganization energy λ_*c*,*m*_, the GS and ES WPs (|χ_*g*_(*t*)⟩ and |χ_*c*_(*t*)⟩, reported here for *t* = 0), and the energy-gap standard deviation σ_*gc*,*m*_.

The molecular Hamiltonian is given by
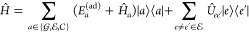
1where *a* runs over three manifolds of electronic states: the ground-state  manifold (that typically only contains a single GS state, *g*), the  manifold in which the nonadiabatic dynamics occurs, and the  manifold of the core-excited states probed by the X-ray probe pulse; *E*_*a*_^(ad)^ is the adiabatic energy of the electronic state *a*.  and  represent the dependence over the nuclear coordinates, and in the LVC model (employing dimensionless coordinates) they read
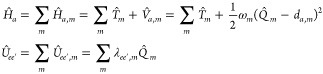
2where *m* runs over the normal modes (accounted for in the LVC model), *a* denotes a generic state of the three manifolds, while *g*, *e*/*e*′, and *c* denote states that belong to the ,  and  manifolds, respectively.  is the kinetic energy operator along mode *m*,^a^ and the potential energy surface (PES) term  is given by a displaced harmonic oscillator (DHO) potential, whose displacement for the electronic state *a* and along the given mode *m* is given by *d*_*a*,*m*_.^b^ We further assume that all electronic states *a* have the same set of normal modes and frequencies (generally computed for the ground state *g*). Without loss of generality, we assume *d*_*g*,*m*_ = 0 ∀ *m*, so that
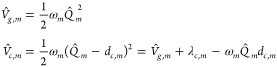
3where *Q*_*m*_ is the normal mode coordinate of mode *m*, *d*_*c*,*m*_ is the displacement (along mode *m*) of the *c* state PES with respect to the ground state PES, and  is the reorganization energy of state *c* along mode *m* (see Figure 1).

 promotes the nonadiabatic dynamics between electronic states of the  manifold. By leveraging on the time-scale separation between the extremely short core-excited-state lifetime and the longer population-transfer time, the term  can be neglected during core excitations. For the same reason, we neglect the nonadiabatic coupling between electronic states of the  manifold. We will consider projections of the Hamiltonian in eq 2 onto various electronic state manifolds, i.e.,
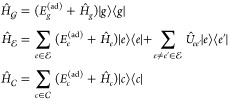
4

We assume that electronic states belonging to different manifolds are coupled only via dipole interactions (i.e., only the external electromagnetic field can promote a change of manifold),^c^ which in the Condon approximation is given by
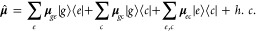
5It is useful here to define some projections of the dipole moment onto specific manifolds, i.e.,
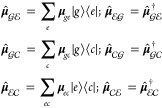
6

By following the steps of ref (1), the molecular wave function at a given time *t* is defined as
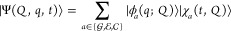
7where  represents the nuclear WP evolving on the *a*-th electronic state  and *q* denotes the electronic degrees of freedom. By employing a *diabatic* formulation, the electronic wave function becomes (ideally) independent of the nuclear coordinates. Moreover, the condition  = 1 holds at all times *t*. The electronic wave function  is conveniently denoted as ,  and  for states in the three manifolds; hereafter, we will drop the explicit coordinate dependence, so that
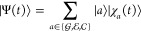
8

Finally, in what follows, we will use the notation *a* → *b* in the subscript of the nuclear WP to denote the transfer process that occurs between the two electronic states *a* and *b*; more precisely,  corresponds to that *fraction* of the nuclear WP initially prepared in *a* at *t* = 0, that is found in *b* at time *t*. This means that at each time,  = , which is the sum over all the WPs that, initially prepared in various electronic states *a* at time *t* = 0, evolved driven by the nonadiabatic dynamics along *t*, and now contribute to shaping the WP of state *b* at time *t*.

#### 2.2.2 Linear Spectra: X-ray Absorption Spectroscopy/XPS

The first-order response function within a QD formulation, employing the WPO approach and without making any assumption besides the Condon approximation, is given by (being interested in the X-ray response, we only consider transitions to  states and neglect those to  ones)^1^
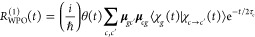
9where  is an exponential decay factor that accounts for the finite-core excited-state lifetime τ_*c*_ (here assumed to be identical for all the states in the  manifold),^d^ which quickly eliminates the *g*–*c*′ coherence;  is the *g*–*c*′ nuclear WP overlap at time *t*. Note that in general both the Gaussian and exponential decay factors can be considered: the second accounts for the finite lifetime, the first, e.g., for the static disorder that could also contribute to the overall spectral broadening.

Assuming the *c* state lifetime to be shorter than the time-scale of the population transfer in the  manifold allows us to neglect *c* → *c*′ transfer process; this means that the dynamics on each initially populated *c* state is purely adiabatic and unaffected by the other states. The double summation in eq 9 reduces to a single summation over the states in the  manifold, and we obtain
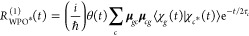
10The * symbol in the WPO* subscript implies that we have assumed that transfer is slow compared to the lifetime,^e^ so that one can consider an adiabatic dynamics that remains in the given state *c* for all *t* times (as indicated by the symbol c*). In what follows for brevity, we set ℏ = 1.

The nuclear overlap factor  can be rewritten as
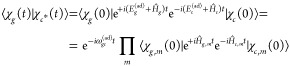
11where , with Δ*E*_*gc*_^(ad)^ being the adiabatic transition energy between states *g* and *c* (that in the DHO model corresponds to the 0–0 energy), and the propagators of the bra and ket WPs appear. Let us focus on one mode, *m*. At zero temperature, the GS WP is prepared in the lowest vibrational level of each mode; furthermore, by assuming impulsive interactions with the external electromagnetic field, we have  =  = , with |*v*_*m*,0_⟩ being the lowest GS vibrational eigenstate along mode *m*. This means that the laser field creates an exact copy of the GS WP in the ES. , with  and is the *m*-th mode frequency. Considering all of that, we get
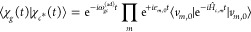
12

There exist different approaches to evaluate the propagator of eq 11, both *time-independent* (expand the WPs in terms of the Hartree product of Harmonic basis functions) and *time-dependent* (compute the overlap explicitly, via a QD simulation, explored in ref (1) in the more general case of nonadiabatic dynamics, i.e., when the *c* → *c*′ transfer could happen). In the present case of adiabatic dynamics, one may write analytical expressions for eq 12 which are exact.^50,51^ These various approaches are summarized in Section S2 of the Supporting Information.

Even if an analytical evaluation of eq 12 is possible, we nonetheless consider a different approach, which is the focus of the present work and set the steps that will be followed in the TA case, where complete analytical expressions are not available (some derivations are reported in Section S2 of the Supporting Information). Let us define two quantities, the average energy of the projected GS WP onto the ES well (along mode *m*), , and the energy-gap variance between states *g* and *c*, σ_*gc*;*m*_^2^. These read, respectively, as
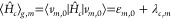
13and
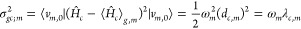
14where these equalities, which hold for the DHO model, admit straightforward generalization to analytical expressions that also include Duschinsky and temperature effects.

Let us go back to the overlap expression of eq 12. By adding and subtracting  to  in the exponent, we get
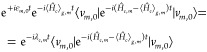
15

Let us now expand the exponential operator in the overlap term in a Taylor series, obtaining
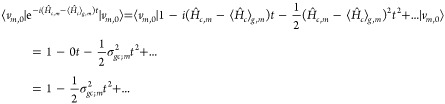
16At this point, we invoke the time-scale separation between vibrational motions on the (core-)excited state and its lifetime: we assume the *c* state lifetime to be shorter than the fastest (spectroscopically relevant) vibration in the system. This allows us to truncate the expansion to second order for short *t*. Equation 16 is then equal to the expansion of a Gaussian to the second order, so that
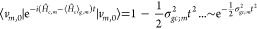
17

Putting all the pieces together finally gives
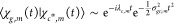
18which is valid in the assumption of time-scale separation between vibrations and the *c* state lifetime.

Considering all *N* modes and following the same steps detailed above for a single mode *m*, one obtains an approximate expression for  in terms of the vertical energy gap and the energy-gap variance, i.e.,
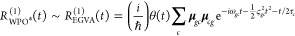
19where the subscript EGVA denotes the *energy-gap/energy-gap variance approximation*, ω_*gc*_ = ω_*gc*_^(ad)^ + ∑_*m*_λ_*c*,*m*_ is the vertical energy-gap frequency, and ς_*gc*_^2^ = ∑_*m*_σ_*gc*;*m*_^2^ is the total variance, i.e., the sum of variances along all modes.

We already noted that an analytical expression for  can be written in terms of line shape functions.^50^ In the adopted notation, this reads
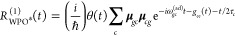
20where *g*_*ab*_(*t*) is the so-called line shape function (whose expression is given in Section S3 of the Supporting Information). Interestingly, the same result of eq 19 can be derived by a second-order Taylor expansion of the line shape function, obtaining  (see the Supporting Information).

In eq 19, all the pieces required for the linear absorption spectrum in the energy gap/energy-gap variance limit become apparent: the center of the transition (the first momentum of the spectrum) is determined by the vertical energy gap ω_*gc*_, while the broadening (the second momentum of the spectrum) is given by the WP energy width term . The lifetime term  further contributes to the line shape. By Fourier transformation of eq 19 along the time *t*, one get a so-called *Voigt* line shape profile. The Voigt profile is symmetric; thus, the derived expressions are not able to describe the asymmetry of the vibronic spectra. This follows from truncating the Taylor series at the second order, i.e., by computing the spectrum employing only its first two moments only. The band asymmetry indeed depends on the higher spectrum moments. Interestingly, the manipulations we have performed are closely related to those introduced to achieve a semiclassical approximation of the LA spectrum,^50,52,53^ and the first two moments are the only two that can be evaluated exactly in such a framework (if the Franck–Condon approximation holds), whereas fully QD approaches are mandatory to compute accurately higher-order moments of the spectrum.^52^

While closing this section, we remark the fact that even taking into account Duschinsky mixings and temperature effects it is possible to derive analytical expressions both for the energy gap and the energy-gap variance^52,54,55^ as well as for the full correlation function (see, e.g., eq 10) whose Fourier transform gives the LA spectrum.^53,56,57^

In the Results and Discussion section, we will compare X-ray absorption spectroscopy (XAS) spectra obtained with the WPO* expression (eq 10), the approximated EGVA expression (eq 19), and the EGA expression, which completely neglects the broadening induced by the WP *width* and is obtained truncating the WP overlap Taylor series to the first order in *t*.

#### 2.2.3 Nonlinear Spectra: TR-XAS/TR-XPS

We consider a TR-XAS experiment, in which the pump pulse (in the vis/UV spectral region) excites the system in the valence manifold of states  (at *t* = 0), and after a time interval *t*_2_ an X-ray probe pulse further excites the system into the core-excited manifold of states . Note that everything applies very similarly also to TR-XPS, for which the  manifold should include core-ionized states instead of core-excited states.

By following similar steps to those that led from eq 10 to eq 19, we now derive the approximate expression for the nonlinear (third-order) response. The main difference is that the WP that is projected (impulsively) by the probe pulse from the  to the  manifold, is no more the cold GS WP: it is rather the QD (nonadiabatically) evolved WP along *t*_2_.

**Figure 2:**
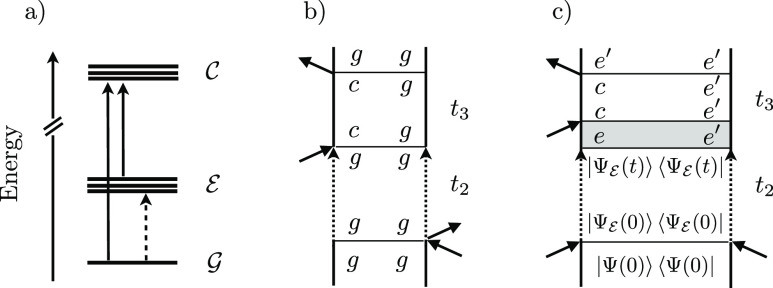
(a) Schematic of the electronic state manifolds and allowed transitions (*pump*: dashed arrow; *probe*: full arrows). (b) Feynman diagram for the GSB contribution. (c) Feynman diagram for the ESA contribution in which the initial conditions are implicitly accounted for in Ψ. In (c), the gray area highlights the fact that  is unpacked in its contributions, and then the various **μ**_*ec*_ transitions and the subsequent |*c*⟩⟨*e*′| coherences are considered.

The third-order response function reads
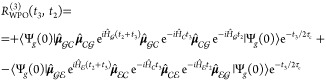
21In writing eq 21, we have removed the common (*i*/ℏ)^3^θ(*t*_2_)θ(*t*_3_) prefactor and we have expressed both Hamiltonian and dipole operators as acting in/between specific manifolds. Note that only the ground-state bleaching (GSB, first term) and the excited-state absorption (ESA, second term) contributions are reported in eq 21, as no stimulated emission is possible in this vis/UV pump–X-ray probe setup. The Feynman diagrams for the GSB and ESA contributions are drawn in Figure 2.

We now express the response function equations in a form that naturally captures the *resulting* effect of the *t*_2_ quantum dynamics without splitting all the pathways over the initial conditions, which is un-necessary. In order to do so, we incorporate the initial dipole–moment interaction in the wave function, so that
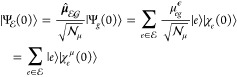
22where , with  being a normalization factor to keep the wave function norm equal to 1; due to the impulsive nature of the laser field, we have that , as previously specified. μ_*ge*_^**ϵ**^ = **μ**_*ge*_·**ϵ**, with **ϵ** being the pump-pulse field polarization. Note that the μ symbol over  is used to keep track of the fact that the WP is prepared in various PESs according to the field–matter interaction. We then rewrite eq 21 as follows
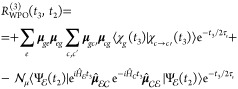
23The GSB contribution closely resembles the first-order contribution presented in the previous section and therefore needs no further discussion. The ESA contribution, instead, requires that the WP, prepared at time *t*_2_ = 0 in (a given electronic state, or in a linear combination of electronic states in) the  manifold, has evolved along *t*_2_. We thus focus on this latter contribution, labeled as *R*_WPO_^(3)ESA^(*t*_3_,*t*_2_).

Let us take a closer look at the *t*_2_ evolved  wave function
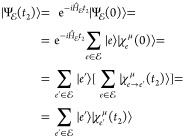
24

**Figure 3:**
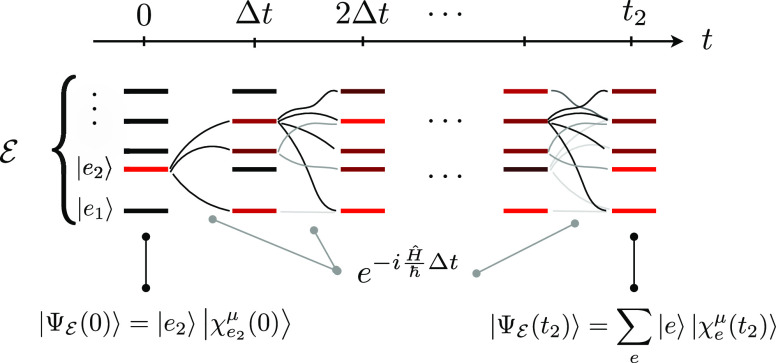
Schematic representation of the WF evolution in the  manifold under the action of the Hamiltonian *Ĥ* that contains nonadiabatic coupling terms between the electronic states *e*. In the example reported in the figure, the wave function is initially prepared in the electronic state *e*_2_ (i.e., *c*_*e*_2__(0) = 1, while all the other amplitudes are set to zero).

Notice that in the last step the effect of the sum over all the initial states  is captured by the *final* amplitudes/WPs in each electronic state , i.e., . The idea here is that the propagation of a wave function prepared in  along *t*_2_ will remain in  but will reshuffle the initially prepared WP, as dictated by the nonadiabatic dynamics, so that every contribution that was on  at time 0 can be redistributed among all other members  of  along *t*_2_, and the final wave function can therefore be written as . See Figure 3 for a pictorial representation of this process.

The ESA contribution to the WPO third-order response therefore reads
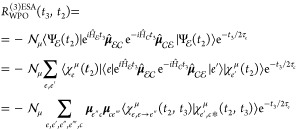
25where we employ a notation that keeps track of the history of the WP: the bra side evolved on the  manifold for the full *t*_2_ + *t*_3_ time interval, while the ket side first evolved in the  manifold along *t*_2_ was projected into the  manifold and thus evolved again in state *c* along *t*_3_.^f^

By invoking the time-scale separation between the lifetime of the *c* state (τ_*c*_, fast) and transfer (slow), transfer along *t*_3_ can be neglected (what we referred to as the WPO* level of theory), and therefore an electronic state (in whatever manifold) will not change along this time interval. The ESA contribution to eq 23 therefore becomes
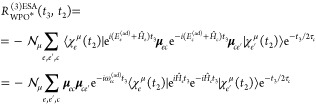
26where the * symbol indicates that we have invoked the above-mentioned time scale separation, for which the dynamics of a WP on a given electronic state is driven only by the Hamiltonian of such state (i.e.,  in this time interval). We have also made use of the fact that the WP on the state *c* are identical to those of *e*′ at *t*_3_ = 0.

To implement the energy-gap approximation, we rewrite eq 26 by splitting *population* and *coherence* contributions (i.e., terms for which the bra and ket WPs are identical or different, respectively), obtaining
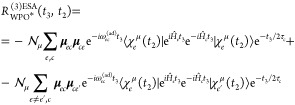
27

We now consider a normalized nuclear WP at each time *t*_2_, i.e., .^g^ The square of the normalization factor has a simple physical interpretation: it gives the probability to find the WP in a given electronic state *e*, i.e., it is the *population* of the electronic state *e*, hereafter referred to as ρ_*e*_(*t*_2_). Therefore, we have
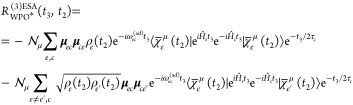
28

We next turn to the bracket term. Let us again consider a single mode *m*; also, we here derived the population contribution (*e* = *e*′): a similar derivation for the coherence contribution (*e* ≠ *e*′) is reported in Section S5 of the Supporting Information.

First, we perform a similar trick previously done, by introducing  and ,^h^ where the *t*_2_ subscript highlights the fact that these quantities depend on *t*_2_ (i.e., on the shape of the WPs on the *e* PESs, just before projecting it onto  states); we then rewrite the exponential operators as
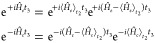
29The constant exponential terms can be moved outside of the bracket; we then perform the Taylor expansion of the exponentials within the ⟨···⟩ symbol, retaining only second-order terms in *t*_3_, which is justified when the separation of time scales between the fast lifetime slow vibrations is invoked.  is then replaced by
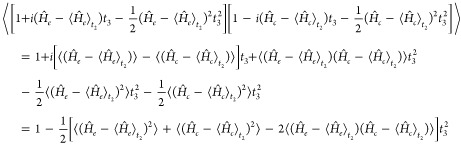
30where in the derivation we have employed the fact that . This is not the case for the coherence contribution. One would be tempted to rewrite the term in front of *t*_3_^2^ as the matrix element of the *e*–*c* energy-gap variance computed on the *t*_2_ evolved WP on both *e* and *e*′ PESs, which is given by
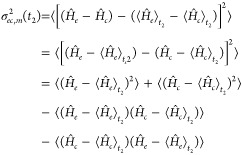
31Nonetheless, this is not the same result reported in eq 30, as , i.e., the two operators do not commute. Therefore, the *t*_3_^2^ term in square brackets in eq 30 does not simplify to the energy gap variance, and we will refer to it as the *pseudo*-variance  (where the *t*_2_ dependence is made explicit), given by
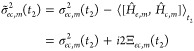
32The commutator term of the pseudovariance turns out to be a purely imaginary *t*_2_ dependent term, here called *i*2Ξ_*ec*,*m*_(*t*_2_), which we demonstrate to be the expectation value of the nuclear momentum along the *m*-th mode (see Section S4 of the Supporting Information for a derivation of such term).

Following the same step performed for the linear response  we get
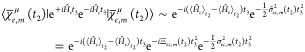
33

The population term of the ESA response function in the EGVA approximation, taking into account all *N* modes, therefore reads
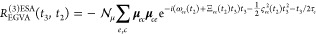
34In the last expression, the relevant terms to be computed are the *t*_2_-dependent energy-gap frequency , the variances ς_*ec*_^2^(*t*_2_), which are the sums of all the (*t*_2_ dependent) *ec*-energy-gap variances along the *N* modes, and the Ξ_*ec*_(*t*_2_) = ∑_*m*_Ξ_*ec*,*m*_(*t*_2_) term, that acts as a linear chirp term along *t*_3_. Notice that
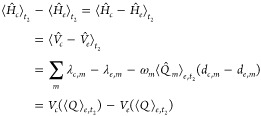
35implying that the energy-gap expectation value can be evaluated as the difference of the two potential energies computed at , where  is the centroid of the WP on state *e* along mode *m*. Similarly, the *t*_2_-dependent energy-gap variance can be recast in terms of the *Q̂* and  matrix elements of the WP on state *e*, i.e.,
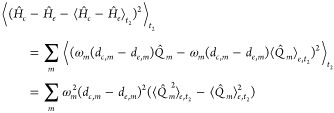
36and the WP coordinate variance now shows up.

Moreover, we have
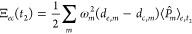
37where  is the expectation value of the momentum operator along mode *m* (see Section S4 of the Supporting Information). The complete pseudovariance expression therefore reads
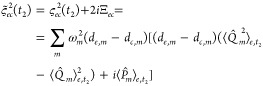
38

A clear physical interpretation of the obtained quantities can be drawn:  is the vibrational contribution to the energy-gap between the *c* and *e* PESs evaluated at the WP centroid and ω_*ec*_(*t*_2_) gives simply the vertical energy-gap frequency computed for each time *t*_2_ (at the WP centroid). As prescribed by eq 36, the energy-gap variance can be obtained through the WP variance along each nuclear coordinate *Q*_*m*_.

To summarize, the EGVA expression of the third-order response, reported in eq 34, allows all quantities to be obtained from a QD only along the *t*_2_ time (i.e., without running QD and WP overlap evaluation along *t*_3_).

## 3 Results and Discussion

In this section, we compare linear and nonlinear spectroscopy simulations of the pyrene molecule in the X-ray window, obtained by employing the LVC/WPO* and LVC/EGVA approaches. Comparison with LVC/WPO is also considered to assess the impact of neglecting the  nonadiabatic dynamics in the short-limit time-scale dictated by τ_*c*_.

An LVC model Hamiltonian of pyrene, parametrized via ab initio CASSCF/CASPT2 inputs, was employed as a benchmark. The lowest 7 excited singlet states were considered in the  manifold, accounting for the dipole moments coupling to the GS, and the nonadiabatic coupling between them. We have considered the internal conversion processes taking place after photoexcitation to the first bright state (S_2_), that mainly involve the transition to the lower dark S_1_ state. The other electronic states serve as mediators of the energy-transfer process, as shown by our previous studies.^31^ Our model was used to run quantum dynamics on pyrene along several nuclear coordinates with the MCTDH method.^1,31^ In order to speed ease the computation of the WP overlaps along the simulations, we used MCTDH and restricted the number of nuclear DOFs to 15 out of a total of 49 that were included in the original ML-MCTDH dynamics,^31^ see ref (1) for details on DOF selection.^i^

First, we report, for a restricted number of transitions (i.e., the brightest ones) the comparison of WPO* and EGVA XAS spectra (obtained as the Fourier transform of the first-order response functions; see expressions reported in eq 39). The total XAS spectrum (that accounts for contributions from all 75 *g* → *c* transitions, with ) is also reported at the EGVA level. We then discuss the results and the appropriateness of the EGVA approximation. In particular, we assessed the quality of the XAS spectra in the EGVA limit for different τ_*c*_’s, and for high- and low-frequency vibrational modes (this last study is reported in Section S6 of the Supporting Information). One expects the EGVA approach to be optimal for short lifetimes (for which the second-order truncation of the overlap Taylor expansion is justified) and for low vibrational frequencies (the lower the mode frequency, the longer the mode period, the better justified the time-scale separation between the lifetime and vibrational motion of the WP).

We then compare the WPO* and EGVA nonlinear (transient absorption) spectra (by Fourier transformation along the time *t*_3_ of the third-order responses reported in eq 40). Note that no coherence ESA contributions need to be computed for pyrene, which simplifies the calculations. Two levels of theory could be considered: the complete EGVA approach, which accounts for the pseudovariance in the response function expressions and for which one needs to evaluate the purely imaginary term *i*2Ξ_*ec*_(*t*_2_) along the *t*_2_ quantum dynamics, and the reduced rEGVA approach, which sets to zero the commutator term and thus only requires computing the (standard) energy-gap variance along the *t*_2_ quantum dynamics. We also examined the accuracy of the approximation of neglecting population transfer in the  manifold along *t*_3_, i.e., when the probe creates a  coherence, by comparing the WPO and WPO* TA spectra.

### 3.1 Linear Absorption XAS Spectrum

We first rewrite the two expressions for the first-order response function, i.e., WPO* and EGVA. While we already highlighted that, in the approximation of neglecting nonadiabatic dynamics, an analytical expression for *R*^(1)^(*t*) can be written,^50^ here we formulate the linear WPO* response function in terms of the time-dependent WP overlap to remain consistent with the definition of the nonlinear response discussed in the next section. Thus, we have
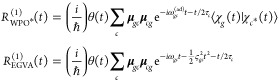
39

#### 3.1.1 WPO* versus EGVA Linear Absorption

**Figure 4:**
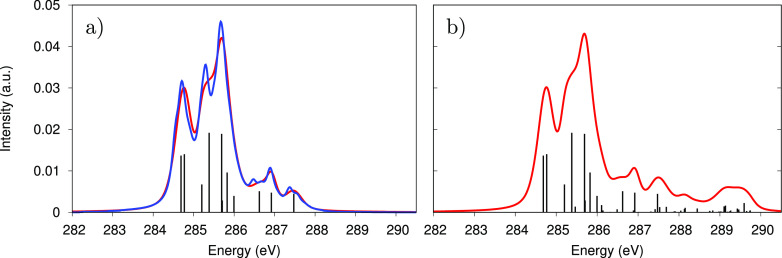
(a) XAS spectrum for the 11 brightest *g* → *c* transitions: EGVA spectrum (red line) and exact spectrum (blue line). (b) Total XAS spectrum for the full set of 75 *g* → *c* transitions, computed only at the EGVA level. In both plots, the stick spectrum is also reported (black bars), and τ_*c*_ is set to 3 fs.

Figure 4a compares the XAS spectrum computed at both levels of theory for the 11 brightest transitions (vertical energy gaps, TDMs, and variances of these transitions are reported in Section S9 of the Supporting Information). The core-excited-state lifetime was set to τ_*c*_ = 3 fs for all states (while we also report results with longer lifetimes, τ_*c*_ = 5 and τ_*c*_ = 7 fs, in Section S7 of the Supporting Information).

The WPO* spectrum is quite accurately reproduced by the EGVA approach with minor differences that can be ascribed to the inability of the EGVA to describe the band asymmetry and possible vibronic side bands.

The total spectrum (comprising 75 transitions) is reported in Figure 4b. Note that, in the high-energy region of the spectrum, a large number of low-intensity transitions accumulate and form an additional shoulder centered at 289.5 eV. We should also note that experimental XAS spectra (not yet available) would also include transitions to quasi/bound and unbound states in the continuum, resulting in a broad and unstructured contribution to the spectrum rising around the carbon K-edge ionization potential. These are not accounted for here.

#### 3.1.2 WPO* versus EGVA Linear Spectra at Various τ_*c*_’s

**Figure 5:**
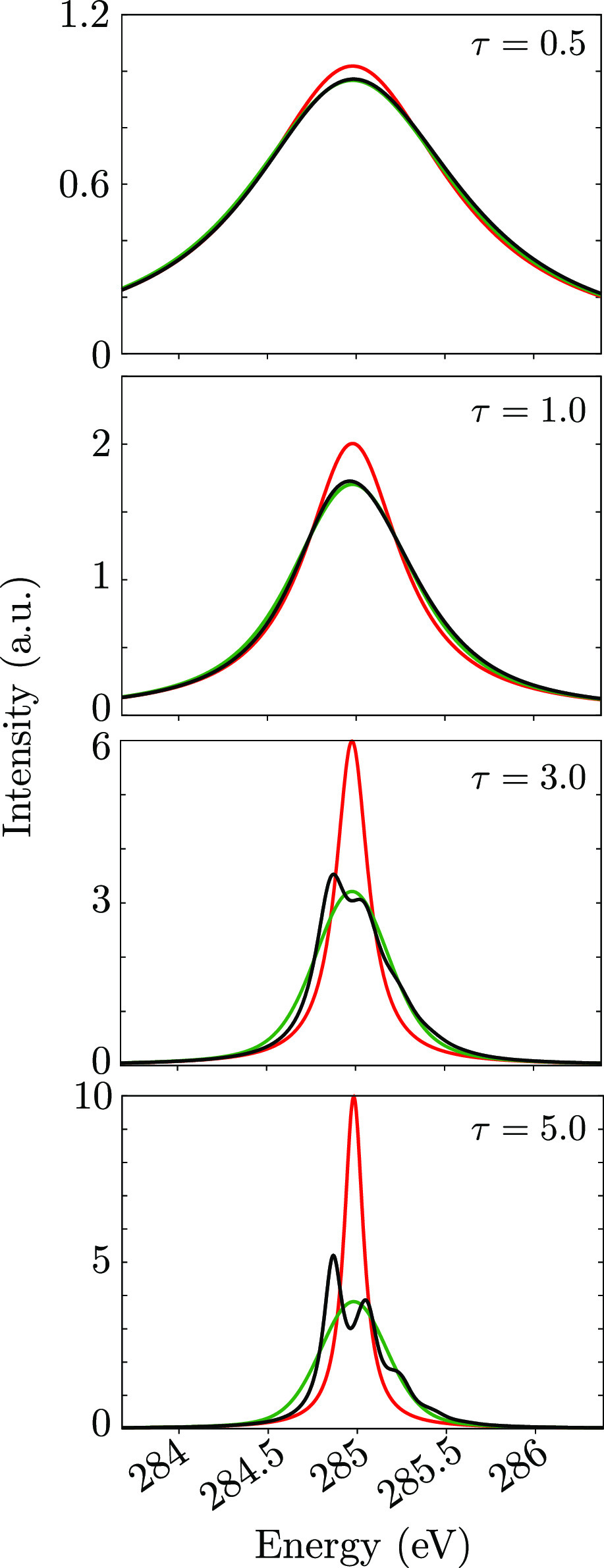
Comparison of carbon K-edge XAS spectra obtained via WPO* (black line), EGVA approximation (green line), and EGA, which simply sets the WP overlap to 1 (red line) and thus gives a Lorentzian line width to the considered transition. Different lifetime values τ_*c*_ (in fs) are considered: as expected, the shorter the lifetime, the higher the quality of the EGVA approximation. Accounting for the overlap via the energy-gap variance greatly improves the spectrum with respect to just setting the overlap term to 1 (EGA). For the considered transition, the vertical energy gap is 284.7 eV, and the energy-gap variance is ς_*gc*_^2^ = 0.026 eV^2^.

Figure 5 compares the WPO*, the EGVA, and the EGA spectra for the *g* → *c*_1_ transition, for different τ_*c*_’s. The EGVA spectrum has a Voigt line shape (Fourier transform of *R*_EGVA_^(1)^(*t*)), while the EGA one possesses a Lorentzian profile (which is what is obtained when setting the WP overlap term to 1, or, equivalently, when truncating the Taylor expansion to the first order in *t*). We note that the EGA/Lorentzian spectrum is always narrower than the other two; the EGVA/Voigt spectrum is much more accurate in capturing the band as a whole and compares well with the WPO* spectrum: indeed, WPO* and EGVA spectra have the same variance. Note also that, as expected, both EGA and EGVA approaches miss the asymmetry of the WPO* band, as both the Lorentzian and Voigt line shapes are symmetric. It is interesting to note how even for the very short lifetimes expected for X-rays, including the variance in the spectrum, greatly improves the line shape with respect to the EGA approach, which means that vibrational broadening is of the same order or larger than the (homogeneous) lifetime broadening already at τ_*c*_ ∼ 2 or 3 fs.

The WPO*/EGVA linear spectra for high- and low-frequency vibrational modes are reported in the Supporting Information (Section S6).

#### 3.1.3 EGVA Transition Width

**Figure 6:**
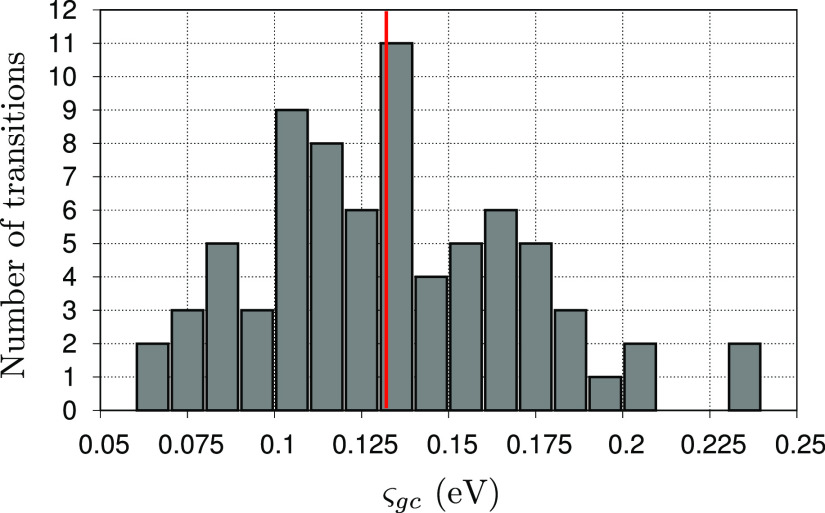
Histogram of ς_*gc*_ values for the 75  transitions that builds the XAS spectrum. The binning size was set to 0.01 eV; the vertical red bar indicates the average ς_*gc*_ value of about 0.13 eV.

In closing this section, we show the extent of the variability of the standard deviation ς_*gc*_ (in eV) for the 75 considered  transitions. In Figure 6, we reported the histogram of the ς_*gc*_ values, employing a binning size of 0.01 eV. The average ς_*gc*_ is at about 0.13 eV with variations between 0.06 and 0.23 eV (which, would it be the only contribution to the broadening would correspond to a band full width at half-maximum of about 0.14 and 0.54 eV, respectively). We note that assigning the same phenomenological broadening to all the transitions would deteriorate the spectrum shape, especially when states of very different nature are present in the spectrum (as, e.g., 1s → π* and 1s → σ* transitions). Figure S11 of the Supporting Information shows the comparison of total XAS obtained employing the calculated ς_*gc*_^2^ values and a constant ς_*gc*_^2^ value (set to the average of all the calculated variances). As expected, the spectrum obtained by employing a constant variance value tends to smooth out some spectral features. This is particularly relevant in TA, where ESA signals may arise from a few relatively isolated bright transitions, therefore showing significant differences if computed with their own variance or a constant phenomenological value.

### 3.2 Transient Absorption

In the previous section, we tested the quality of the EGVA approximation for the static XAS spectrum. We now turn to transient spectroscopy: having the possibility of avoiding the *t*_3_ propagation of the WP in the  manifolds and to compute the *t*_3_-dependent overlap, while still accounting for the nonadiabatic dynamics during *t*_2_ evolution of the wave function at the quantum dynamics level, is the main goal of our approach which exploits the EGVA approximation.

**Figure 7:**
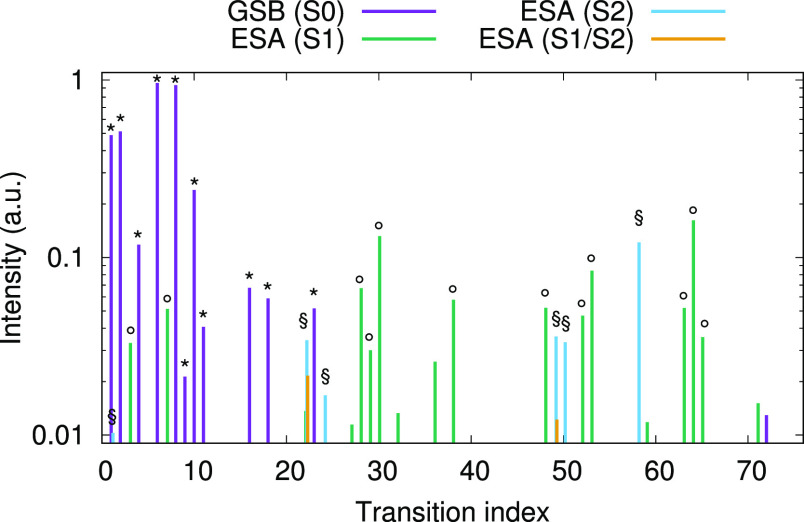
Intensities from products of dipoles for various transient absorption contributions (**μ**_*ac*_ **μ**_*ca*′_, with *a* and *a*′ being equal to *g*, S_1_ and/or S_2_). GSB (core excitations from *g*) are depicted in violet; ESA (population) contributions from S_1_ are depicted in green; ESA (population) contributions from S_2_ (less intense than the former) are depicted in cyan; ESA (coherence) contributions that involve both S_1_ and S_2_ are depicted in orange. Note that the spectra are dominated by population contributions: coherence ESA contributions are therefore neglected. The symbols *, ◦, and § denote the transitions (from *g*, S_1_, and S_2_, respectively) that have been selected for the evaluation of the spectra via the overlap method.

For pyrene transient absorption (TR-XAS), we consider the S_2_ → S_1_ internal conversion process: the pump is assumed to be resonant only to the bright S_2_ state (and the system initial state—after the pump interaction—is ). Moreover, pyrene has no core-excited states *c* that are simultaneously dipole-coupled to both S_2_ and S_1_ (see Figure 7): therefore, the coherence term of eq 28 can be dropped which results in the following working equations
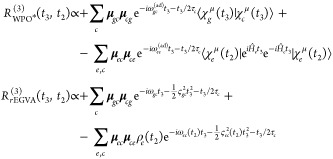
40In both formulations, the first term is the GSB term (which again in the WPO* formulation can be evaluated analytically) and the second is the ESA term. Moreover, for simplicity, we have removed the common (*i*/ℏ)^3^θ(*t*_2_)θ(*t*_3_) factor, as well as the common  normalization factor.

To assess the validity of the approximation, we compare results for the WPO and WPO* expressions, which explicitly compute the overlap, with the equations obtained employing the energy-gap/energy-gap variance approximation without computing the commutator term, a level of theory that we refer to as *reduced* EGVA or rEGVA. The states were selected by a brightness criterion. A total of 28 core-excited states have been considered: 11 dipole coupled to *g*, 12 dipole coupled to S_1_, and 6 dipole coupled to S_2_ vertical energy gaps, and TDMs are given in the Supporting Information (Section S9).

**Figure 8:**
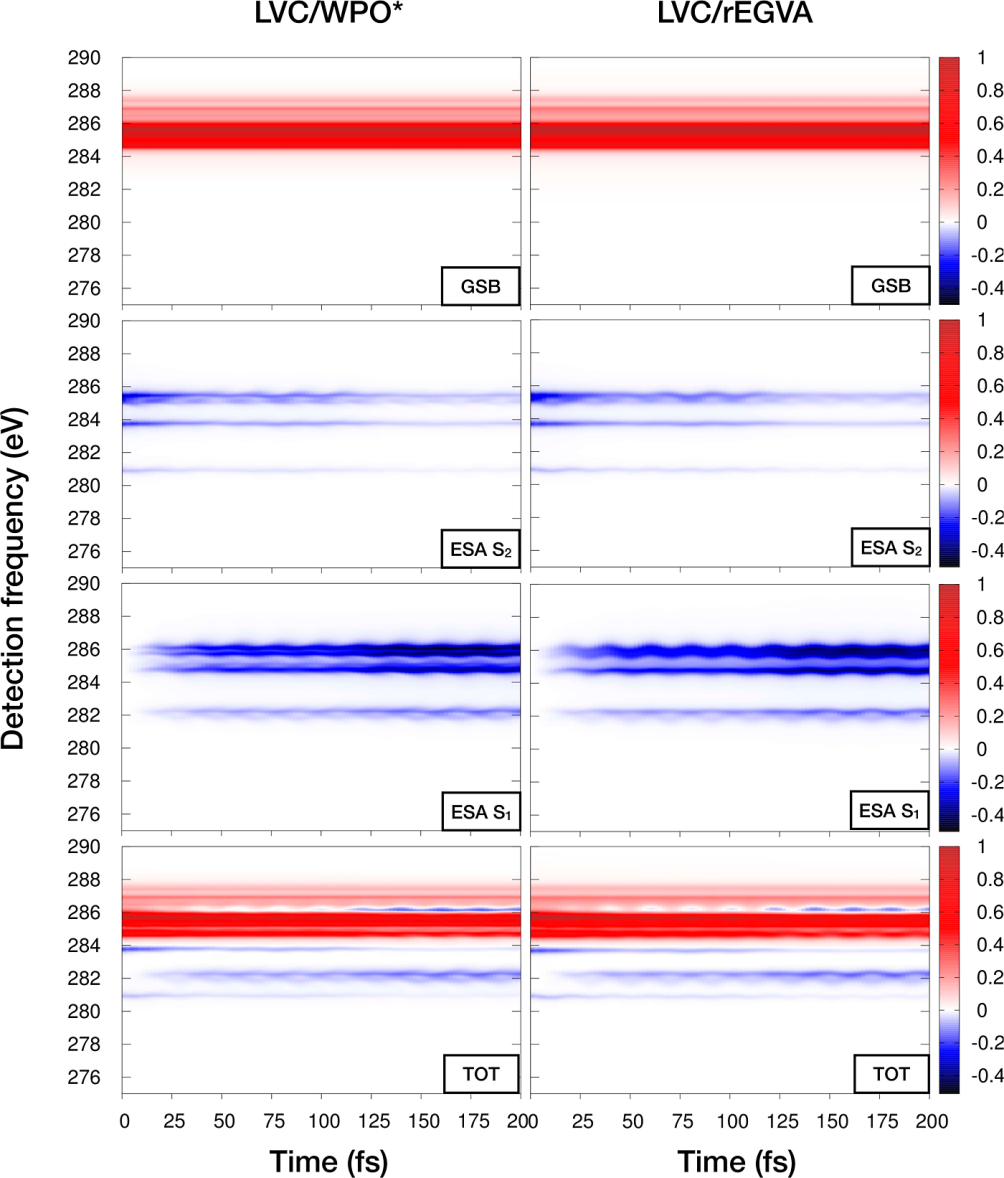
Comparison of WPO* (*left*) and rEGVA (*right*) TA spectra for the *brightest* transitions. The total spectrum and various contributions (GSB, ESA from S_1_, and ESA from S_2_) are shown. The core-excited-state lifetime is set to τ_*c*_ = 3 fs.

Figure 8 shows the WPO* and rEGVA TA spectra. The agreement is good, even though the standard variance has been used instead of the prescribed pseudovariance. This suggests that in the present system neglecting the purely imaginary term *i*Ξ_*ec*_(*t*_2_) does not significantly affect the results. Note that the energy-gap variation along *t*_2_ captures the oscillatory behavior of the signals along the detection frequency axis at different *t*_2_ times. The GSB signal covers almost half of the considered window. Nonetheless, the spectrum shows a background-free region around 280–283 eV in which the dying out of the S_2_ → *c* signals and the concomitant growing of the S_1_ → *c* ones are clearly observed; therefore, these signals can be used to follow the photoinduced dynamics on the  manifold.

**Figure 9:**
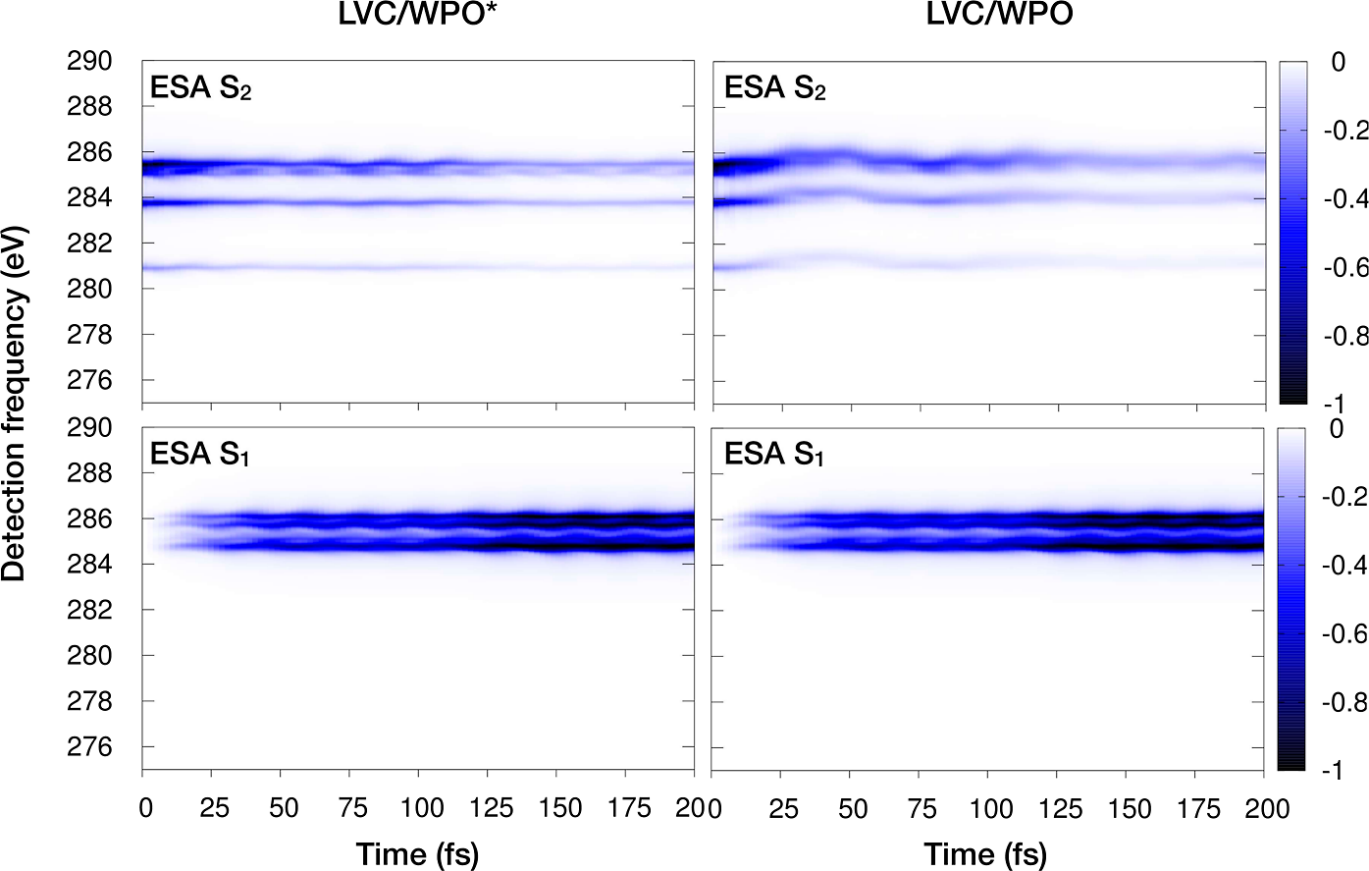
Comparison of selected WPO* (*left*) and WPO (*right*) ESA contributions in the TA. In WPO*, population transfer is assumed to occur on longer time scales than the core-excited-state lifetime (here set to τ = 3 fs) and therefore the nonadiabatic dynamics in the  manifold is switched off along the *t*_3_ time. The WPO level of theory considers population transfer in the  at all times. Only slight deviations are noted in the S_2_ ESA signals (with WPO signals generally broader than the WPO* ones and with a slight blue shift of the former signal with respect to the latter level of theory), while an extremely accurate reproduction is shown for S_1_ ESA.

To assess the impact of neglecting nonadiabatic dynamics along *t*_3_, in Figure 9, we compare WPO and WPO* TA spectra. The main difference between the two approach is that in WPO one does not switch off the nonadiabatic coupling in the  manifold along *t*_3_. Interestingly, as noted in ref (1), the nonadiabatic dynamics along *t*_3_ affects the shape of the WP on the S_2_ surface, so that ESA signals from S_2_ experience a slight blue shift and a clear enhancement of the band broadening. At variance, the WPO S_1_ ESA signals are completely reproduced at the WPO* level.

**Figure 10:**
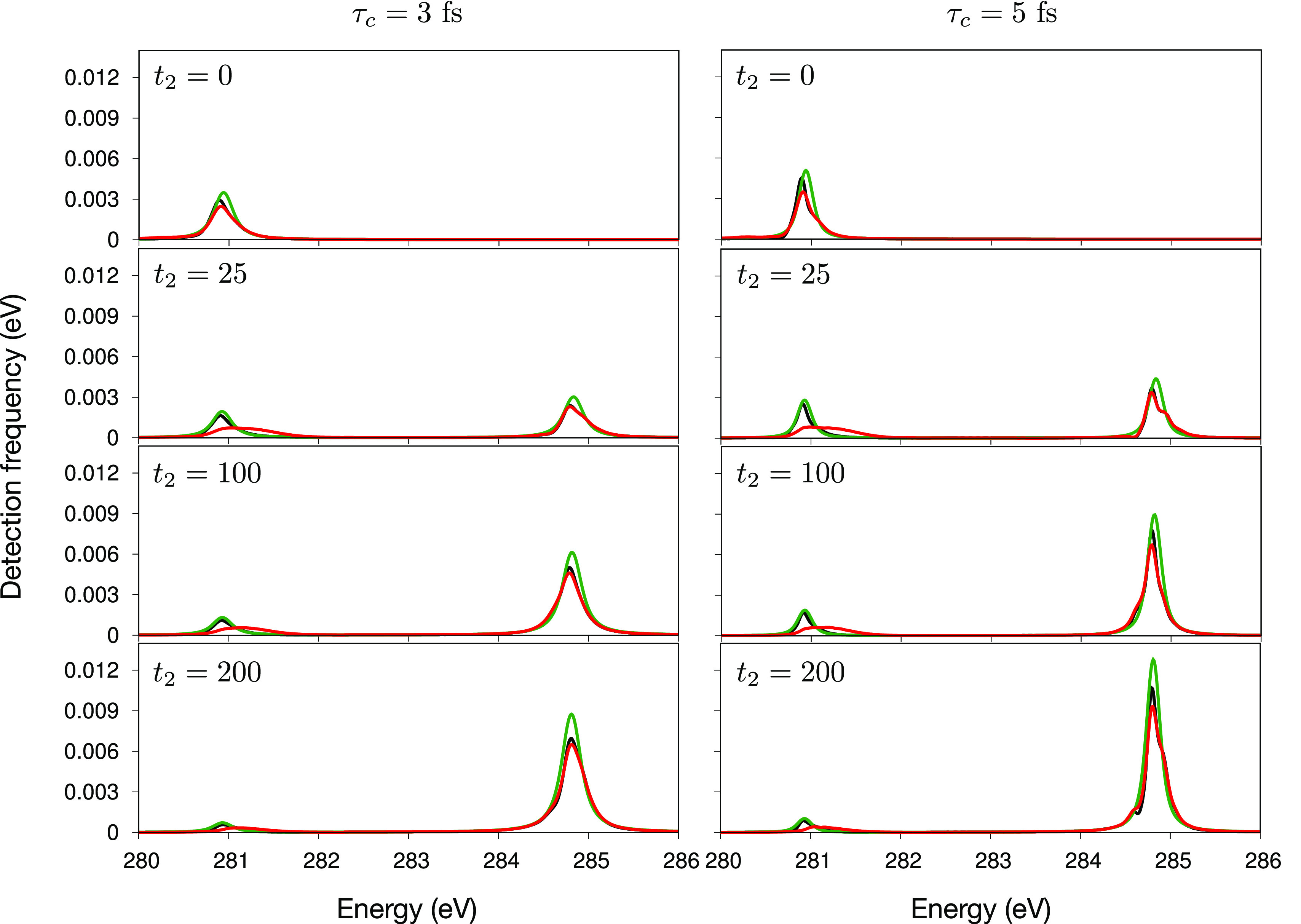
Comparison of TA cuts at selected *t*_2_ times and for different values of τ_*c*_. Only two transitions (ESA) are considered: S_2_ → *c*_30_ (on the left-hand side of the spectrum) and S_1_ → *c*_1_ (on the right-hand side of the spectrum). WPO, WPO*, and rEGVA levels of theory are depicted with red, black, and green curves, respectively. Note that we reported the ESA signal multiplied by −1.

To get additional insight, in Figure 10, we study cuts of the WPO, WPO* and rEGVA TA spectra for two transitions (namely, S_2_ → *c*_30_, which appears on the low-energy side of the spectrum, and S_1_ → *c*_1_, on the high-energy side of the spectrum) at selected *t*_2_ times: *t*_2_ = 0 fs (where all the population is on S_2_), *t*_2_ = 25 and 100 fs (where the population is split between S_2_ and S_1_), and *t*_2_ = 200 fs (where most of the population eventually accumulates on S_1_). A few relevant observations are as follows: as expected, the S_2_ → *c*_30_ ESA diminishes with time, while the S_1_ → *c*_1_ ESA grows, which mirrors the population dynamics; WPO and WPO* can assume an asymmetric line shape with vibronic side bands, while rEGVA (as well as EGVA) miss these finer details; finally, the impact of the nonadiabatic dynamics along *t*_3_ is clearly assessed by comparing WPO and WPO* cuts of the S_2_ → *c*_30_ ESA signal which is considerably broader at the WPO level.

**Figure 11:**
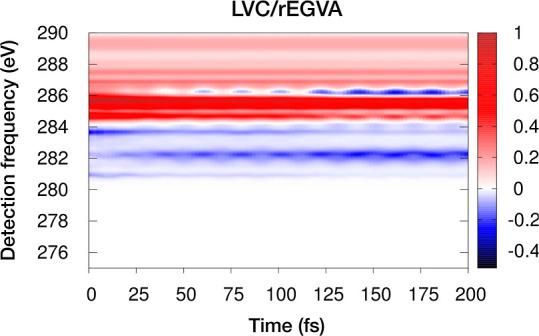
TA spectrum obtained at the rEGVA level, considering all the transitions from the valence-excited states (*g*, S_1_, and S_2_) to all of the core-excited states (*c*_1_–*c*_75_). Only population contributions have been computed. τ_*c*_ = 3 fs.

Figure 11 shows the rEGVA TA map computed by including all of the transitions obtained from quantum chemistry calculations (and not just the brightest ones). The spectrum closely resembles the total spectrum in Figure 8.

To conclude, we note that the (reduced) 15-mode model considered here, which was fine-tuned to describe the dynamics in the  manifold, might have left-out modes that are relevant to describe the spectroscopy of the core-excited states, such as strongly displaced modes along some of the  states. Therefore, spectroscopy simulations of a full-mode model might show some differences, with the left-out modes possibly contributing to the additional broadening/vibronic structure of the transitions. This is clearly illustrated in Section S10 of the Supporting Information, where we compare the *g* → *c*_1_ linear absorption spectrum in the reduced (15 modes) and the full-mode model. To overcome this issue, we mention that a strategy that mixes numerical QD propagation along a restricted number of modes with analytical evaluation of the remaining (*spectroscopically relevant*) modes has been recently reported in ref (58).

## 4 Conclusions

An efficient approach to simulate nonlinear X-ray spectroscopy has been demonstrated. Approximate expressions that exploit the accuracy of quantum dynamics along the interpulse waiting time *t*_2_ while taking advantage of the extremely short lifetime of core-excited/ionized states along the detection time *t*_3_ were derived by means of Taylor expanding the WP propagator in time and truncating at the second order. The approximation is physically motivated by the time-scale separation between states’ lifetime τ_*c*_, which is assumed to be shorter than any other relevant dynamics time scale (i.e., that of electronic population dynamics and that of nuclear vibrational motion). This allows omission of explicit propagation of the WP on both the valence- and core-excited states manifold along *t*_3_ as done in the exact protocol termed WPO. The obtained expressions require computing a few relevant quantities only along the *t*_2_ dynamics in the manifold of valence-excited states: the energy gap at the centroid of the evolving WP and the energy-gap pseudovariance (EGVA) or standard variance (*r*EGVA, or reduced EGVA). Our protocol positions itself between the EGA, which neglects the energy-gap variance, and the exact WPO protocols, as it incorporates a higher degree of physical detail than EGA without significant additional computational demand (essentially requiring a QD simulation only in the manifold of valence states) but is unable to recover finer details such as asymmetrical broadening and vibronic structures seen in WPO simulations which can be only recovered by considering terms beyond the quadratic in the Taylor expansion, with an increase of the computational cost.

We presented XAS and TR-XAS for the ultrafast S_2_ → S_1_ internal conversion in pyrene at both the LVC/WPO and approximate LVC/*r*EGVA levels of theory, showing excellent agreement that validates the approximation underlying the *r*EGVA. The limits of the time-scale separation and, thus the validity of the *r*EVGA, were explored by analyzing the dependence of the spectral line shapes on the core-excited state lifetimes τ_*c*_, as well as on the mode frequencies. Following the validation of the approximation, complete TR-XAS at the carbon K-edge was simulated at the LVC/*r*EGVA level, thereby incorporating into the Hamiltonian nearly 100 core-excited states. The spectra reveal signatures of the S_2_ disappearing with *t*_2_ accompanied by emerging signatures of the S_1_ state accumulating the excited-state population in a background free spectral window between 280 and 284 eV. The signals are modulated by oscillatory features due to the coherent vibrational motion in the excited states. Despite not having an experimental counterpart, we are confident that the simulated spectra will agree with future TR-XAS experiments, which will be the ultimate validation of separating the time scales on which core-excited states decay and nuclei move for the purpose of accelerating simulations in the X-ray regime.

## Supporting Information Available

Active spaces for core-excited states; different approaches to simulate the LA spectrum; line shape functions second-order Taylor expansion; the commutator ; the coherence *R*_WPO*_^(3)ESA^(*t*_3_,*t*_2_) term in the EGVA approximation; WPO* vs EGVA LA: high- and low-frequency modes; WPO* vs EGVA LA at various values of τ_*c*_; EGVA XAS spectrum: variable vs constant variance; energy gaps, energy-gap variances, and TDMs; pyrene LA—full-mode models vs reduced-mode model; and response function Fourier transform—technical details (PDF)

## Footnotes

a In dimensionless coordinates, it reads , with  being the momentum operator along the *m*th coordinate.

b Note that such *d*_*a*,*m*_ displacements can be obtained either at the Franck–Condon geometry (simply computing the *a*-th state gradient, which, along the given mode *m*, is given by −ω_*m*_*d*_*a*,*m*_) or via an excited-state geometry optimization. Here, we employ the (computationally cheaper) first approach. The two results are equivalent only when the surfaces are perfectly harmonic and obey the DHO model.

c If, on the time scale of interest, the process that brings an excited electronic state back to the ground electronic state is relevant, one may add a term in the Hamiltonian that couples these two manifolds.

d Note that one could in principle consider different τ_*c*_ values for dissociative and nondissociative core-excited states.

e Note that the WPO* first-order response function of eq 10 can be evaluated analytically by means of line shape functions.^1^

f Note that the most general formulation would also consider the *c* → *c*′ transfer process. We still assume that accounting for such a process is immaterial as, in the dense manifold of core-excited states, the nonadiabatic dynamics is fast, and its effect can be accounted for by further shortening the lifetime term τ_*c*_.

g Note that such a procedure is correct only when ⟨χ_*e*_^μ^|χ_*e*_^μ^⟩ ≠ 0. At early times, for dark states, this might not be the case.

h Note that we simplify the notation: it is understood that the ⟨···⟩ symbol refers to that specific matrix element; using a similar but more elaborate notation, one could have specified that by writing , with *a* = *b* or *c*. Since this is clear from the context, we will use the simpler notation.

i Even though the total number of pyrene normal modes is 72, for symmetry reasons, 23 of them have zero coupling and gradient, so that only 49 are required.

## References

[ref1] Segatta F.; Ruiz D. A.; Aleotti F.; Yaghoubi M.; Mukamel S.; Garavelli M.; Santoro F.; Nenov A. Nonlinear Molecular Electronic Spectroscopy via MCTDH Quantum Dynamics: From Exact to Approximate Expressions. J. Chem. Theory Comput. 2023, 19, 2075–2091. 10.1021/acs.jctc.2c01059. 36961952 PMC10100531

[ref2] Paz Y. Transient IR spectroscopy as a tool for studying photocatalytic materials. J. Phys.: Condens. Matter 2019, 31, 503004 10.1088/1361-648X/ab3eda. 31469092

[ref3] Maiuri M.; Garavelli M.; Cerullo G. Ultrafast Spectroscopy: State of the Art and Open Challenges. J. Am. Chem. Soc. 2020, 142, 3–15. 10.1021/jacs.9b10533. 31800225

[ref4] Conti I.; Cerullo G.; Nenov A.; Garavelli M. Ultrafast Spectroscopy of Photoactive Molecular Systems from First Principles: Where We Stand Today and Where We Are Going. J. Am. Chem. Soc. 2020, 142, 16117–16139. 10.1021/jacs.0c04952. 32841559 PMC7901644

[ref5] Pellegrini C.; Marinelli A.; Reiche S. The physics of x-ray free-electron lasers. Rev. Mod. Phys. 2016, 88, 015006 10.1103/RevModPhys.88.015006.

[ref6] Zhao Z.; Wang D.; Gu Q.; Yin L.; Gu M.; Leng Y.; Liu B. Status of the SXFEL Facility. Appl. Sci. 2017, 7, 607 10.3390/app7060607.

[ref7] Boutu W.; Ducousso M.; Hergott J.-F.; Merdji H. Optical Technologies for Extreme-Ultraviolet and Soft X-ray Coherent Sources; Canova F., Poletto L., Eds.; Springer: Berlin, Germany, 2008; p 63.

[ref8] Calegari F.; Sansone G.; Stagira S.; Vozzi C.; Nisoli M. Advances in attosecond science. J. Phys. B: At., Mol. Opt. Phys. 2016, 49, 062001 10.1088/0953-4075/49/6/062001.

[ref9] Chen L. X.; Zhang X.; Shelby M. L. Recent advances on ultrafast X-ray spectroscopy in the chemical sciences. Chem. Sci. 2014, 5, 4136–4152. 10.1039/C4SC01333F.

[ref10] Milne C.; Penfold T.; Chergui M. Recent experimental and theoretical developments in time-resolved X-ray spectroscopies. Coord. Chem. Rev. 2014, 277–278, 44–68. Following Chemical Structures using Synchrotron Radiation 10.1016/j.ccr.2014.02.013.

[ref11] Chergui M.; Collet E. Photoinduced Structural Dynamics of Molecular Systems Mapped by Time-Resolved X-ray Methods. Chem. Rev. 2017, 117, 11025–11065. 10.1021/acs.chemrev.6b00831. 28692268

[ref12] Bhattacherjee A.; Leone S. R. Ultrafast X-ray Transient Absorption Spectroscopy of Gas-Phase Photochemical Reactions: A New Universal Probe of Photoinduced Molecular Dynamics. Acc. Chem. Res. 2018, 51, 3203–3211. 10.1021/acs.accounts.8b00462. 30462481

[ref13] Young L.; Ueda K.; Gühr M.; Bucksbaum P. H.; Simon M.; Mukamel S.; Rohringer N.; Prince K. C.; Masciovecchio C.; Meyer M.; et al. Roadmap of ultrafast x-ray atomic and molecular physics. J. Phys. B: At., Mol. Opt. Phys. 2018, 51, 032003 10.1088/1361-6455/aa9735.

[ref14] Kraus P. M.; Zürch M.; Cushing S. K.; Neumark D. M.; Leone S. R. The ultrafast X-ray spectroscopic revolution in chemical dynamics. Nat. Rev. Chem 2018, 2, 82–94. 10.1038/s41570-018-0008-8.

[ref15] Geneaux R.; Marroux H. J. B.; Guggenmos A.; Neumark D. M.; Leone S. R. Transient absorption spectroscopy using high harmonic generation: a review of ultrafast X-ray dynamics in molecules and solids. Philos. Trans. R. Soc., A 2019, 377, 20170463 10.1098/rsta.2017.0463. PMC6452051 30929624

[ref16] List N. H.; Dempwolff A. L.; Dreuw A.; Norman P.; Martínez T. J. Probing competing relaxation pathways in malonaldehyde with transient X-ray absorption spectroscopy. Chem. Sci. 2020, 11, 4180–4193. 10.1039/D0SC00840K. 34122881 PMC8152795

[ref17] Scutelnic V.; Tsuru S.; Pápai M.; Yang Z.; Epshtein M.; Xue T.; Haugen E.; Kobayashi Y.; Krylov A. I.; Møller K. B.; Coriani S.; Leone S. R. X-ray transient absorption reveals the 1Au (nπ*) state of pyrazine in electronic relaxation. Nat. Commun. 2021, 12, 5003 10.1038/s41467-021-25045-0. 34408141 PMC8373973

[ref18] Kaczun T.; Dempwolff A. L.; Huang X.; Gelin M. F.; Domcke W.; Dreuw A. Tuning UV Pump X-ray Probe Spectroscopy on the Nitrogen K Edge Reveals the Radiationless Relaxation of Pyrazine: Ab Initio Simulations Using the Quasiclassical Doorway–Window Approximation. J. Phys. Chem. Lett. 2023, 14, 5648–5656. 10.1021/acs.jpclett.3c01018. 37310800

[ref19] Keefer D.; Schnappinger T.; de Vivie-Riedle R.; Mukamel S. Visualizing conical intersection passages via vibronic coherence maps generated by stimulated ultrafast X-ray Raman signals. Proc. Natl. Acad. Sci. U.S.A. 2020, 117, 24069–24075. 10.1073/pnas.2015988117. 32929028 PMC7533881

[ref20] Keefer D.; Aleotti F.; Rouxel J. R.; Segatta F.; Gu B.; Nenov A.; Garavelli M.; Mukamel S. Imaging conical intersection dynamics during azobenzene photoisomerization by ultrafast X-ray diffraction. Proc. Natl. Acad. Sci. U.S.A. 2021, 118, 118 10.1073/pnas.2022037118. PMC7826416 33436412

[ref21] Nam Y.; Keefer D.; Nenov A.; Conti I.; Aleotti F.; Segatta F.; Lee J. Y.; Garavelli M.; Mukamel S. Conical Intersection Passages of Molecules Probed by X-ray Diffraction and Stimulated Raman Spectroscopy. J. Phys. Chem. Lett. 2021, 12, 12300–12309. 10.1021/acs.jpclett.1c03814. 34931839

[ref22] Beck M. H.; Jäckle A.; Worth G. A.; Meyer H.-D. The multiconfiguration time-dependent Hartree (MCTDH) method: a highly efficient algorithm for propagating wavepackets. Phys. Rep. 2000, 324, 1–105. 10.1016/S0370-1573(99)00047-2.

[ref23] Multidimensional Quantum Dynamics: MCTDH Theory and Applications; Meyer H.-D., Gatti F., Worth G. A., Eds.; Wiley VCH: Weinheim, 2009.

[ref24] Gatti F.; Lasorne B.; Meyer H.-D.; Nauts A. Lecture Notes in Chemistry; Springer International Publishing, 2017; Vol. 98.

[ref25] Köppel H.; Domcke W.; Cederbaum L. The Multi-mode vibronic-coupling approach. Conical Intersections; Electronic Structure, Dynamics and Spectroscopy; World Scientific, 2004; pp 323–368.

[ref26] Worth G. A.; Meyer H.-D.; Köppel H.; Cederbaum L. S.; Burghardt I. Using the MCTDH wavepacket propagation method to describe multimode non-adiabatic dynamics. Int. Rev. Phys. Chem. 2008, 27, 569–606. 10.1080/01442350802137656.

[ref27] Zuehlsdorff T. J.; Shedge S. V.; Lu S.-Y.; Hong H.; Aguirre V. P.; Shi L.; Isborn C. M. Vibronic and Environmental Effects in Simulations of Optical Spectroscopy. Annu. Rev. Phys. Chem. 2021, 72, 165–188. 10.1146/annurev-physchem-090419-051350. 33395546

[ref28] Anda A.; De Vico L.; Hansen T.; Abramavičius D. Absorption and Fluorescence Lineshape Theory for Polynomial Potentials. J. Chem. Theory Comput. 2016, 12, 5979–5989. 10.1021/acs.jctc.6b00997. 27759961

[ref29] Köppel H. Diabatic Representation: Methods for the Construction of Diabatic Electronic State. Conical Intersections; Electronic Structure, Dynamics and Spectroscopy; World Scientific, 2004; pp 175–204.

[ref30] Yaghoubi Jouybari M.; Liu Y.; Improta R.; Santoro F. Ultrafast Dynamics of the Two Lowest Bright Excited States of Cytosine and 1-Methylcytosine: A Quantum Dynamical Study. J. Chem. Theory Comput. 2020, 16, 5792–5808. 10.1021/acs.jctc.0c00455. 32687360

[ref31] Aleotti F.; Aranda D.; Yaghoubi Jouybari M.; Garavelli M.; Nenov A.; Santoro F. Parameterization of a linear vibronic coupling model with multiconfigurational electronic structure methods to study the quantum dynamics of photoexcited pyrene. J. Chem. Phys. 2021, 154, 104106 10.1063/5.0044693. 33722019

[ref32] Andersson K.; Malmqvist P.-Å.; Roos B. O. Second-order perturbation theory with a complete active space self-consistent field reference function. J. Chem. Phys. 1992, 96, 1218–1226. 10.1063/1.462209.

[ref33] Sauri V.; Serrano-Andrés L.; Shahi A. R. M.; Gagliardi L.; Vancoillie S.; Pierloot K. Multiconfigurational Second-Order Perturbation Theory Restricted Active Space (RASPT2) Method for Electronic Excited States: A Benchmark Study. J. Chem. Theory Comput. 2011, 7, 153–168. 10.1021/ct100478d. 26606229

[ref34] Lee S.-Y.; Pollard W.; Mathies R. A. Quantum theory for transition state absorption. Chem. Phys. Lett. 1989, 160, 531–537. 10.1016/0009-2614(89)80058-2.

[ref35] Freibert A.; Mendive-Tapia D.; Huse N.; Vendrell O. Femtosecond x-ray absorption spectroscopy of pyrazine at the nitrogen K-edge: on the validity of the Lorentzian limit. J. Phys. B: At., Mol. Opt. Phys. 2021, 54, 244003 10.1088/1361-6455/ac3846.

[ref36] Penfold T. J.; Pápai M.; Rozgonyi T.; Møller K. B.; Vankó G. Probing spin–vibronic dynamics using femtosecond X-ray spectroscopy. Faraday Discuss. 2016, 194, 731–746. 10.1039/C6FD00070C. 27711829

[ref37] Capano G.; Milne C. J.; Chergui M.; Rothlisberger U.; Tavernelli I.; Penfold T. J. Probing wavepacket dynamics using ultrafast x-ray spectroscopy. J. Phys. B: At., Mol. Opt. Phys. 2015, 48, 214001 10.1088/0953-4075/48/21/214001.

[ref38] Tsuru S.; Vidal M. L.; Pápai M.; Krylov A. I.; Møller K. B.; Coriani S. Time-resolved near-edge X-ray absorption fine structure of pyrazine from electronic structure and nuclear wave packet dynamics simulations. J. Chem. Phys. 2019, 151, 124114 10.1063/1.5115154. 31575192

[ref39] Foggi P.; Pettini L.; Santa I.; Righini R.; Califano S. Transient absorption and vibrational relaxation dynamics of the lowest excited singlet state of pyrene in solution. J. Phys. Chem. 1995, 99, 7439–7445. 10.1021/j100019a029.

[ref40] Raytchev M.; Pandurski E.; Buchvarov I.; Modrakowski C.; Fiebig T. Bichromophoric Interactions and Time-Dependent Excited State Mixing in Pyrene Derivatives. A Femtosecond Broad-Band Pump-Probe Study. J. Phys. Chem. A 2003, 107, 4592–4600. 10.1021/jp027356c.

[ref41] Neuwahl F. V. R.; Foggi P. Direct Observation of S2–S1 Internal Conversion in Pyrene by Femtosecond Transient Absorption. Laser Chem. 1999, 19, 375–379. 10.1155/1999/37692.

[ref42] Nenov A.; Giussani A.; Fingerhut B. P.; Rivalta I.; Dumont E.; Mukamel S.; Garavelli M. Spectral lineshapes in nonlinear electronic spectroscopy. Phys. Chem. Chem. Phys. 2015, 17, 30925–30936. 10.1039/C5CP01167A. 26084213

[ref43] Roos M. K.; Reiter S.; de Vivie-Riedle R. Ultrafast relaxation from 1La to 1Lb in pyrene: a theoretical study. Chem. Phys. 2018, 515, 586–595. 10.1016/j.chemphys.2018.08.002.

[ref44] Montorsi F.; Segatta F.; Nenov A.; Mukamel S.; Garavelli M. Soft X-ray Spectroscopy Simulations with Multiconfigurational Wave Function Theory: Spectrum Completeness, Sub-eV Accuracy, and Quantitative Reproduction of Line Shapes. J. Chem. Theory Comput. 2022, 18, 1003–1016. 10.1021/acs.jctc.1c00566. 35073066 PMC8830047

[ref45] Fdez Galván I.; Vacher M.; Alavi A.; Angeli C.; Aquilante F.; Autschbach J.; Bao J. J.; Bokarev S. I.; Bogdanov N. A.; Carlson R. K.; et al. OpenMolcas: From Source Code to Insight. J. Chem. Theory Comput. 2019, 15, 5925–5964. 10.1021/acs.jctc.9b00532. 31509407

[ref46] Aquilante F.; Autschbach J.; Baiardi A.; Battaglia S.; Borin V. A.; Chibotaru L. F.; Conti I.; De Vico L.; Delcey M.; Fdez Galván I.; et al. Modern quantum chemistry with [Open]Molcas. J. Chem. Phys. 2020, 152, 214117 10.1063/5.0004835. 32505150

[ref47] Li Manni G.; Fdez Galván I.; Alavi A.; Aleotti F.; Aquilante F.; Autschbach J.; Avagliano D.; Baiardi A.; Bao J. J.; Battaglia S.; et al. The OpenMolcas Web: A Community-Driven Approach to Advancing Computational Chemistry. J. Chem. Theory Comput. 2023, 19, 6933–6991. 10.1021/acs.jctc.3c00182. 37216210 PMC10601490

[ref48] Ma D.; Li Manni G.; Gagliardi L. The generalized active space concept in multiconfigurational self-consistent field methods. J. Chem. Phys. 2011, 135, 044128 10.1063/1.3611401. 21806111

[ref49] Worth G. Quantics: A general purpose package for quantum molecular dynamics simulations. Comput. Phys. Commun. 2020, 248, 107040 10.1016/j.cpc.2019.107040.

[ref50] Mukamel S. Principles of Nonlinear Optical Spectroscopy; Oxford University Press: New York, 1995.

[ref51] Abramavicius D.; Palmieri B.; Voronine D. V.; Šanda F.; Mukamel S. Coherent Multidimensional Optical Spectroscopy of Excitons in Molecular Aggregates Quasiparticle versus Supermolecule Perspectives. Chem. Rev. 2009, 109, 2350–2408. 10.1021/cr800268n. 19432416 PMC2975548

[ref52] Lax M. The Franck-Condon Principle and Its Application to Crystals. J. Chem. Phys. 1952, 20, 1752–1760. 10.1063/1.1700283.

[ref53] Lami A.; Santoro F. Computational Strategies for Spectroscopy; Barone V., Ed.; John Wiley & Sons, Inc., 2011; Chapter 10, pp 475–516.

[ref54] Markham J. J. Interaction of Normal Modes with Electron Traps. Rev. Mod. Phys. 1959, 31, 956–989. 10.1103/RevModPhys.31.956.

[ref55] Biczysko M.; Bloino J.; Santoro F.; Barone V. Computational Strategies for Spectroscopy; Barone V., Ed.; John Wiley & Sons, Inc., 2011; Chapter 8, pp 361–443.

[ref56] Ianconescu R.; Pollak E. Photoinduced Cooling of Polyatomic Molecules in an Electronically Excited State in the Presence of Dushinskii Rotations. J. Phys. Chem. A 2004, 108, 7778–7784. 10.1021/jp037739q.

[ref57] Huh J.; Berger R. Coherent state-based generating function approach for Franck-Condon transitions and beyond. J. Phys.: Conf. Ser. 2012, 380, 012019 10.1088/1742-6596/380/1/012019.

[ref58] Montorsi F.; Aranda D.; Garavelli M.; Santoro F.; Segatta F. Spectroscopy from quantum dynamics: a mixed wave function/analytical line shape functions approach. Theor. Chem. Acc. 2023, 142, 108 10.1007/s00214-023-03035-3.

