# Supplementary material for: Time-Resolved X-ray Absorption Spectroscopy: An MCTDH Quantum Dynamics Protocol
Source: J Chem Theory Comput. 2023 Dec 15;20(1):307–22. doi: 10.1021/acs.jctc.3c00953 (PMC10782456; doi:10.1021/acs.jctc.3c00953)
Supplement: Supplementary file 1 — ct3c00953_si_001.pdf [file ct3c00953_si_001.pdf]

SUPPORTING INFORMATION FOR

Time-resolved X-ray absorption spectroscopy: an MCTDH quantum dynamics protocol

Francesco Segatta,[†] Daniel Aranda,[‡,¶] Flavia Aleotti,[†] Francesco Montorsi,[†] Shaul Mukamel,[§] Marco Garavelli,[*,†] Fabrizio Santoro,[*,¶] and Artur Nenov[*,†]

†*Dipartimento di Chimica Industriale "Toso Montanari", University of Bologna, Viale del Risorgimento, 4, 40136 Bologna, Italy*

‡*ICMol, Universidad de Valencia, c/Catedrático José Beltrán, 2, 46980 Paterna, Spain*

¶*Istituto di Chimica dei Composti Organometallici (ICCOM-CNR), Area della Ricerca del CNR, Via Moruzzi 1, I-56124 Pisa, Italy*

§*Department of Chemistry and Department of Physics and Astronomy, University of California, Irvine, 92697, USA*

E-mail: marco.garavelli@unibo.it; fabrizio.santoro@pi.iccom.cnr.it; artur.nenov@unibo.it

This PDF includes:

- Active spaces for core-excited state;
- Different approaches to simulate the LA spectrum;
- Lineshape functions second order Taylor expansion;
- The commutator $\left[\hat{H}_e, \hat{H}_c\right]$;
- The *coherence* $R^{(3)ESA}_{WPO^*}(t_3, t_2)$ term in the EGVA approximation;
- WPO* vs EGVA LA: high and low frequency modes;
- WPO* vs EGVA LA: at various values of τ_c;
- EGVA XAS spectrum: variable vs constant variance;
- Energy gaps, energy-gap variances and TDMs;
- Pyrine LA: full mode models vs reduced (15) modes model;
- Response function Fourier transform: technical details.

S1 Active spaces for core-excited states

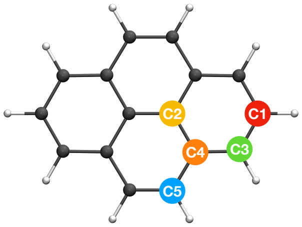

Figure s1: Labeling of the five types of C atoms (multiplicity: 2 for C1 and C2, 4 for C3-C5) identified in the S_0 minimum of pyrene (D_{2h} point group).

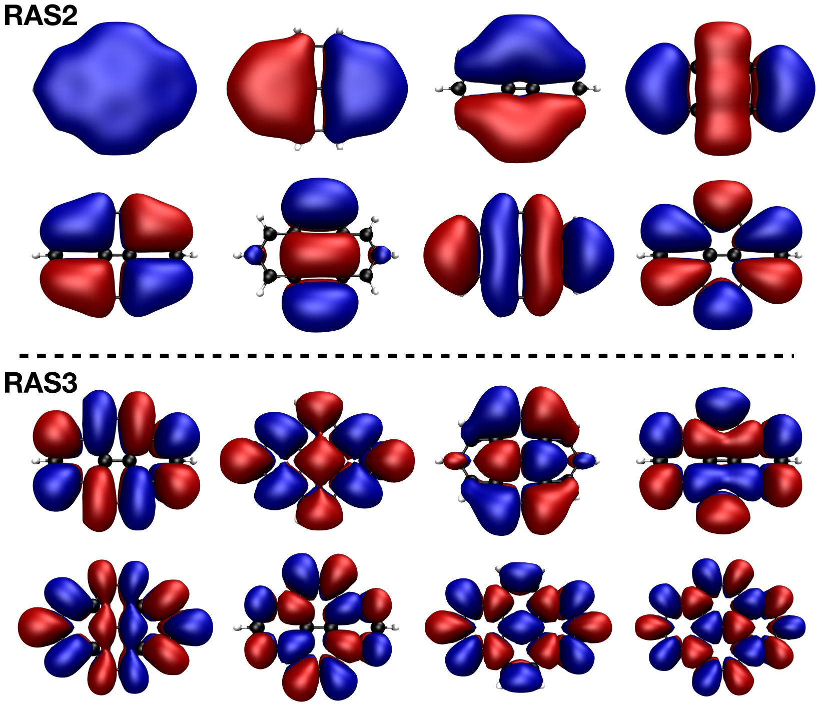

Figure s2: π (RAS2) and π^* (RAS3) orbitals used for the RASSCF/ANO-RCC calculation of the valence excited states (e manifold). For each carbon atom type identified in Figure s1 the active space was completed by putting the corresponding 1s orbital in RAS1.

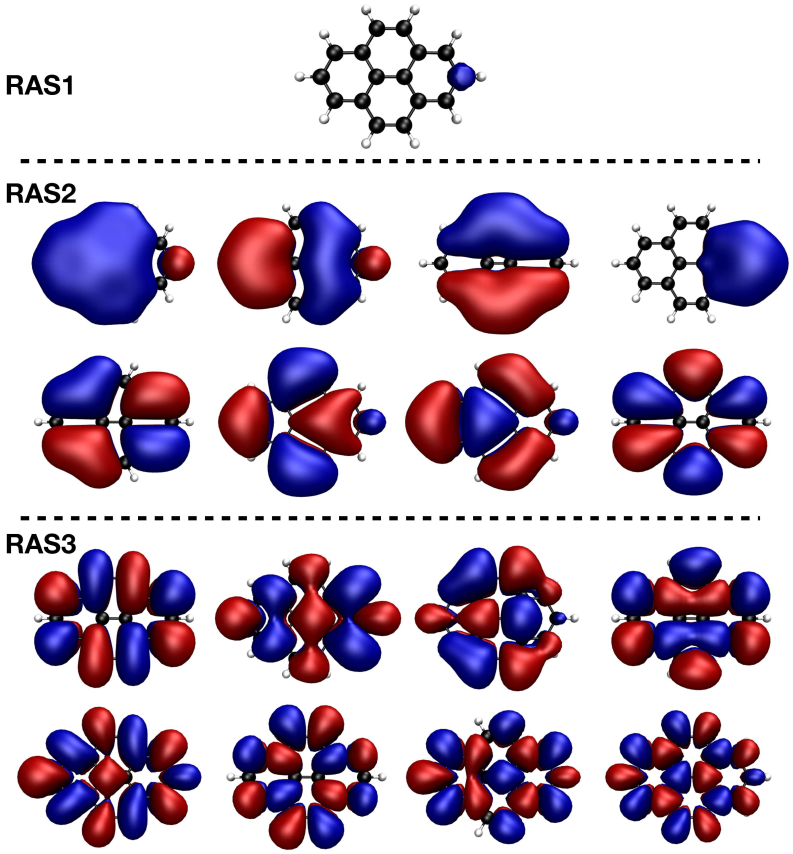

Figure s3: 1s (RAS1), π (RAS2) and π^* (RAS3) orbitals used for the RASSCF/ANO-RCC calculation of the core excited states associated with carbon atom type C1 (see labels in Figure s1).

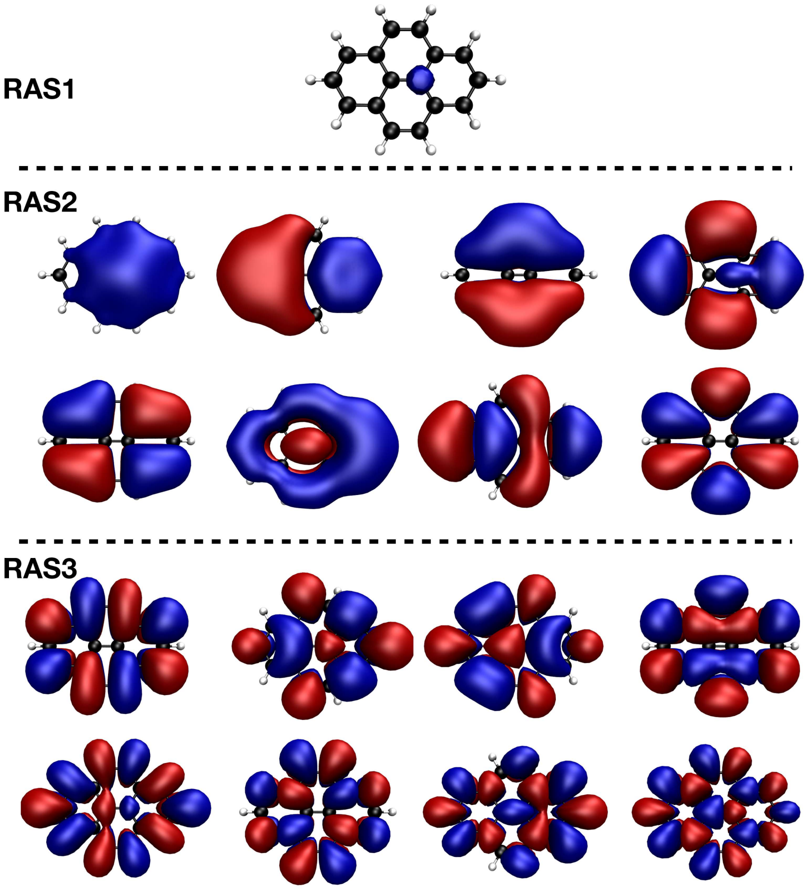

Figure s4: 1s (RAS1), π (RAS2) and π^* (RAS3) orbitals used for the RASSCF/ANO-RCC calculation of the core excited states associated with carbon atom type C2 (see labels in Figure s1).

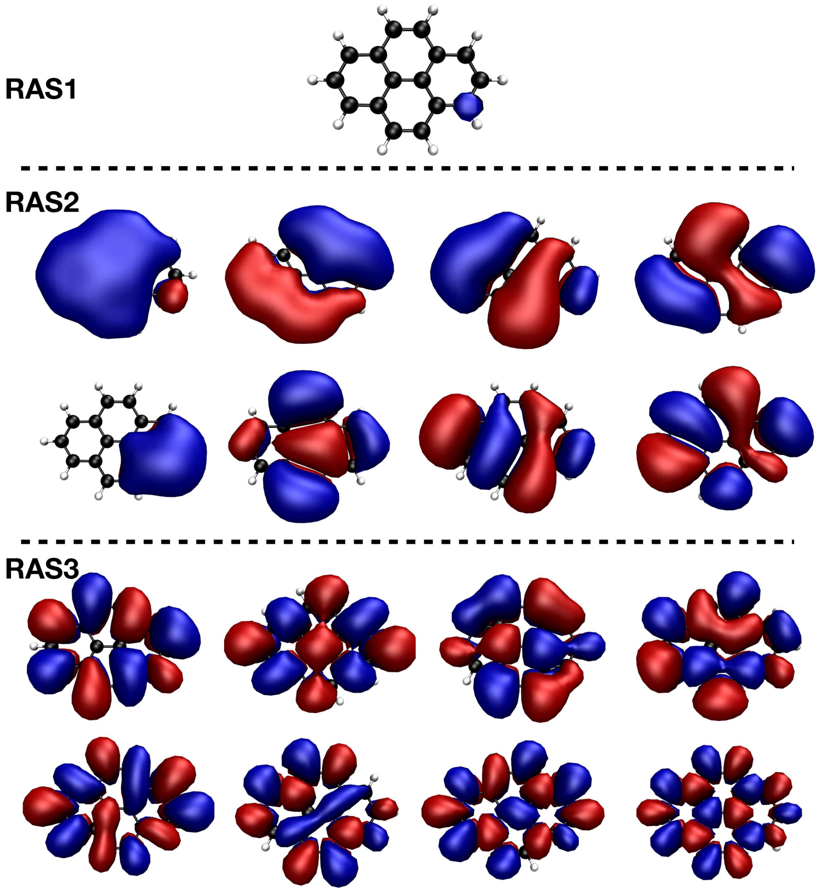

Figure s5: 1s (RAS1), π (RAS2) and π^* (RAS3) orbitals used for the RASSCF/ANO-RCC calculation of the core excited states associated with carbon atom type C3 (see labels in Figure s1).

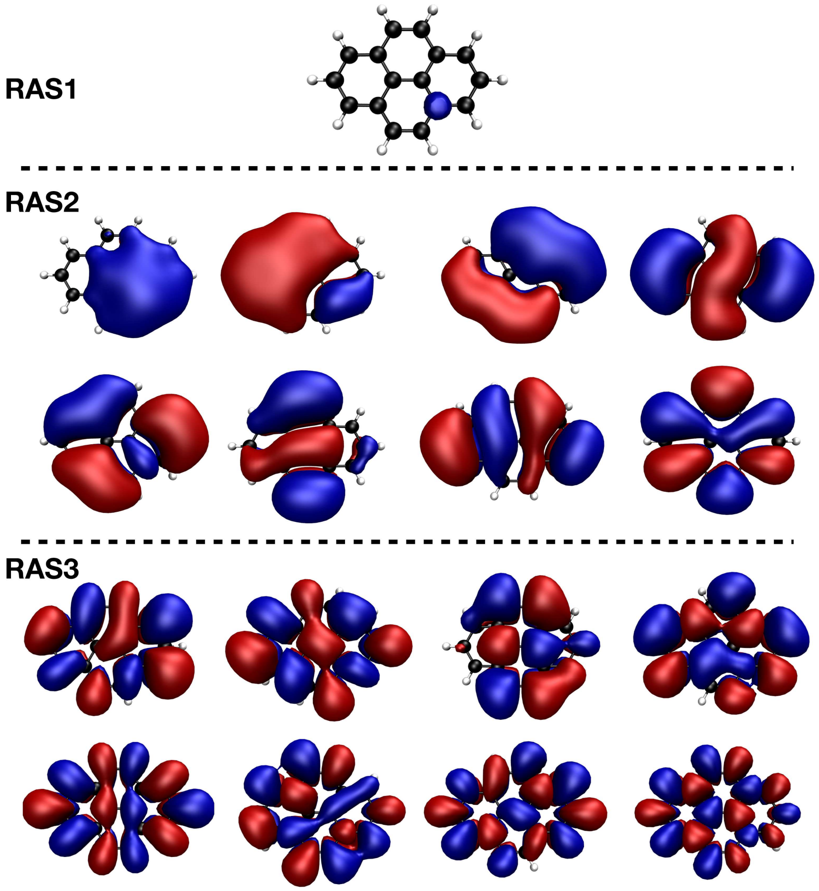

Figure s6: 1s (RAS1), π (RAS2) and π^* (RAS3) orbitals used for the RASSCF/ANO-RCC calculation of the core excited states associated with carbon atom type C4 (see labels in Figure s1).

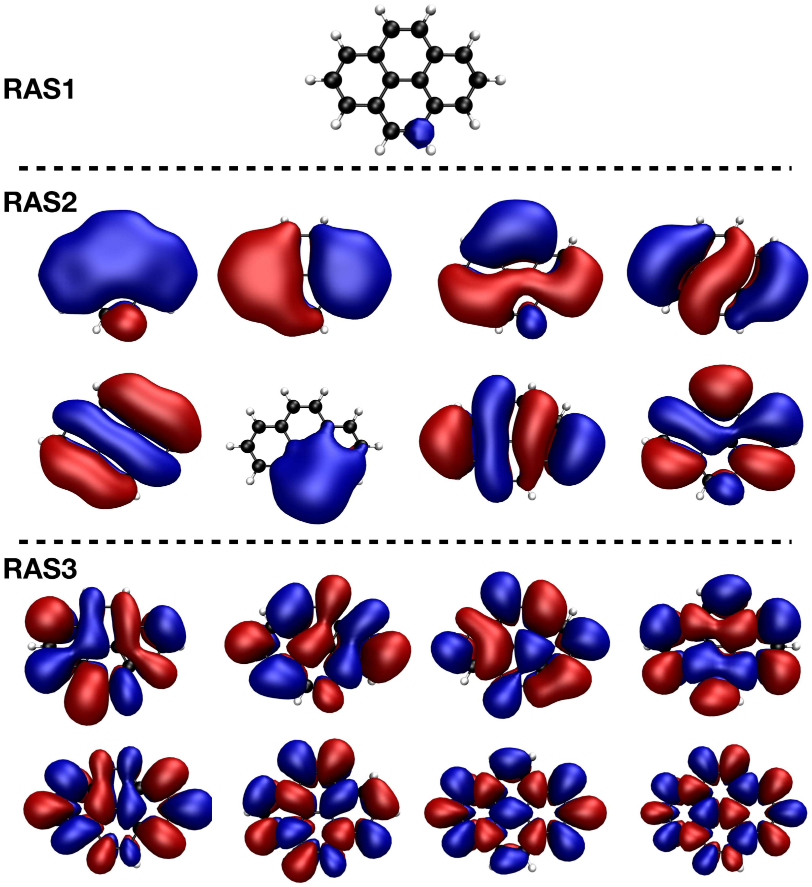

Figure s7: 1s (RAS1), π (RAS2) and π^* (RAS3) orbitals used for the RASSCF/ANO-RCC calculation of the core excited states associated with carbon atom type C5 (see labels in Figure s1).

S2 Different approaches to simulate the LA spectrum

We seek to evaluate the time-dependent overlap term given by

$$\langle \chi_g(t)| \, \chi_{c^*}(t)\rangle = \langle \chi_g(0)| \, e^{+i\hat{H}_g t} e^{-i\hat{H}_c t} \, |\chi_c(0)\rangle \tag{S1}$$

where the $*$ indicates that the ket WP will evolve adiabatically on the potential energy surface of the state c (i.e., it will stay on the electronic state onto which it was projected at time $t = 0$).

S2.1 *Time-independent* approach

We introduce the eigenstates of $\hat{H}_g$ and $\hat{H}_c$, which are the vibrational states of the two wells. We refer to the GS vibrational eigenstates as $|v_{m,i}\rangle$ (where the index m denotes the mode, and the index i denotes the vibrational level on that mode), and the excited state eigenstates as $|u_{m,i}\rangle$. Therefore

$$\hat{H}_g \, |v_{m,i}\rangle = \epsilon_{m,i} \, |v_{m,i}\rangle \tag{S2}$$

$$\hat{H}_c \, |u_{m,i}\rangle = \epsilon_{m,i} \, |u_{m,i}\rangle \tag{S3}$$

$\epsilon_{m,i}$ is the vibrational energy of state $|v_{m,i}\rangle$ (and $|u_{m,i}\rangle$), i.e. $\epsilon_{m,k} = \frac{1}{2}\hbar\omega_m + k\hbar\omega_m$. One can jump from the GS vibrational basis to the ES vibrational basis by a simple basis transformation, given by:

$$|v_{m,i}\rangle = \sum_j \langle u_{m,j}|v_{m,i}\rangle \, |u_{m,j}\rangle \tag{S4}$$

Therefore, for our single mode m, we have $|\chi_{g,m}(0)\rangle = |\chi_{c,m}(0)\rangle = |v_{m,0}\rangle$ (where we have also added the subscript m to the GS and ES WP to specify that this part of the WP is along mode m; the complete WP is the Hartree product $|\chi_g(0)\rangle = \prod_m |\chi_{g,m}(0)\rangle$).[1]

[1]From this it follows that the overlap may be factorized into a product of contributions of each mode, since the total Hamiltonian can be written as a sum of commuting mode-specific Hamiltonians.

We can now return to our original problem, which is to derive an expression for the overlap term as a function of t (again in absence of transport). We first recast the overlap in the form:

$$\langle \chi_{g,m}(0)| e^{+i\hat{H}_g t} e^{-i\hat{H}_c t} |\chi_{c,m}(0)\rangle = \langle v_{m,0}| e^{+i\hat{H}_g t} e^{-i\hat{H}_c t} |v_{m,0}\rangle = e^{+i\frac{\epsilon_{m,0}}{\hbar}t} \langle v_{m,0}| e^{-i\hat{H}_c t} |v_{m,0}\rangle \quad \text{(S5)}$$

where in the last step we employed the fact that $|v_{m,0}\rangle$ is an eigenstate of $\hat{H}_g$, and $\frac{\omega_m}{2} = \frac{\epsilon_{m,0}}{\hbar}$. The problem then turns into the evaluation of $\langle v_{m,0}| e^{-i\hat{H}_c t} |v_{m,0}\rangle$.

We can now expand the bra and ket sides in terms vibrational eigenstates of the ES well, which gives:

$$\begin{aligned}\langle v_{m,0}| e^{-i\hat{H}_c t} |v_{m,0}\rangle &= \sum_{jk} \langle v_{m,0}|u_{m,j}\rangle \langle u_{m,j}|e^{-i\hat{H}_c t}|u_{m,k}\rangle \langle u_{m,k}|v_{m,0}\rangle \\ &= \sum_{k} |\langle v_{m,0}|u_{m,k}\rangle|^2 e^{-i\frac{\epsilon_{m,k}}{\hbar}t} \quad \text{(S6)}\end{aligned}$$

where in the last step we took advantage of the orthogonality of the vibrational states of the ES. Note that the Franck-Condon factor, $|\langle v_{m,0}|u_{m,k}\rangle|^2$ appears in the expression. We finally get a weighted sum of imaginary exponentials, where each exponential oscillates with the frequency of the respective vibrational state, and is weighted by the projection of the initial GS WP onto the ES set of vibrational states.

S2.2 *Time-dependent* (WPO) approach

In Ref. 1 we explored how the time-dependent overlap can be computed explicitly via a QD simulation. This involves propagating the two wave-packets on the g and c surfaces, and computing their overlap at regular time intervals (which are set by the fastest time-scale considered). The interested reader can refer to Ref. 1 for additional details.

S2.3 *Lineshape functions* approach

Mukamel et al. have shown how, in absence of transport (i.e., when the dynamics is adiabatic), this overlap term can be computed analytically. Briefly, one expresses the ES potential in terms of the GS one, rewrites the expression in terms of time-ordered exponential, expand to second-order (the so-called *cumulant expansion*) and obtain the energy fluctuation autocorrelation functions, spectral density, and eventually the line-shape functions.[2,3]

Note that an approach that combines explicit WP overlap calculations along some modes, with analytical expressions via cumulant expansion has been recently demonstrated in Ref. 4.

S2.4 *Energy-gap/Energy-gap variance* approach

The main equations are reported in the main text. Here we provide additional details.

Definition of energy-gap and energy-gap variance

$$\begin{aligned}\langle\hat{H}_c\rangle_{g,m} &= \langle v_{m,0}|\hat{H}_c|v_{m,0}\rangle = \\ &= \langle v_{m,0}|\hat{T}+\hat{V}_c|v_{m,0}\rangle = \\ &= \langle v_{m,0}|\hat{T}+\hat{V}_g+\lambda_{c,m}-\omega_m\hat{Q}_m d_{c,m}|v_{m,0}\rangle \\ &= \langle v_{m,0}|\hat{H}_g|v_{m,0}\rangle+\lambda_{c,m}-\omega_m d_{c,m}\langle v_{m,0}|\hat{Q}_m|v_{m,0}\rangle \\ &= \epsilon_{m,0}+\lambda_{c,m}\end{aligned} \tag{S7}$$

where $\langle v_{m,0}|\hat{Q}_m|v_{m,0}\rangle$ (the centroid of the initial WP) is null. By adding the adiabatic $g-c$ energy gap, $\Delta E_{gc}^{(ad)}$ to this expression, one obtains:

$$\langle\hat{H}_c\rangle_{g,m}+\Delta E_{gc}^{(ad)} = (\Delta E_{gc}^{(ad)}+\lambda_{c,m})+\epsilon_{m,0} = \Delta E_{gc}+\epsilon_{m,0} \tag{S8}$$

where ΔE_{gc} is the vertical transition, which summed with the zero point energy $\epsilon_{m,0}$ gives the first moment of the spectrum.

$$
\begin{aligned}
\sigma^2_{gc;m} &= \langle v_{m,0}|(\hat{H}_c - \langle \hat{H}_c \rangle_{g,m})^2 | v_{m,0}\rangle = \\
&= \langle v_{m,0}| \left(\hat{T} + \hat{V}_g + \lambda_{c,m} - \omega_m \hat{Q}_m d_{c,m}\right)^2 |v_{m,0}\rangle - (\epsilon_{m,0} + \lambda_{c,m})^2 = \\
&= \omega_m^2 \left(d_{c,m}\right)^2 \langle \hat{Q}_m^2 \rangle_{g,m} = \\
&= \frac{1}{2}\omega_m^2 \left(d_{c,m}\right)^2 = \\
&= \omega_m \lambda_{c,m}
\end{aligned}
\tag{S9}
$$

where in the last equality we made use of the fact that $\langle \hat{Q}_m^2 \rangle_{g,m} = 1/2$ (in dimensionless coordinates). $\sigma^2_{gc;m}$ is easily recognized as the energy-gap variance: in fact,

$$
\begin{aligned}
\sigma^2_{gc;m} &= \langle v_{m,0}|(\hat{H}_c - \langle \hat{H}_c \rangle_{g,m})^2 | v_{m,0}\rangle = \\
&= \langle v_{m,0}| \left(\left[\hat{H}_c + \Delta E^{(ad)}_{gc}\right] - \left[\langle \hat{H}_c \rangle_{g,m} + \Delta E^{(ad)}_{gc}\right]\right)^2 |v_{m,0}\rangle
\end{aligned}
\tag{S10}
$$

would is the (vertical) energy-gap variance.

WP overlap: from one mode, to all modes

$$
\begin{aligned}
\langle \chi_g(t)|\chi_c(t)\rangle &= e^{-i\omega^{(ad)}_{gc} t} \prod_m \langle \chi_{g,m}(0)| e^{+i\hat{H}_{g,m} t} e^{-i\hat{H}_{c,m} t} |\chi_{c,m}(0)\rangle \sim \\
&\sim e^{-i\omega^{(ad)}_{gc} t} \prod_m e^{-i\lambda_{c,m} t} e^{-\frac{1}{2}\sigma^2_{gc;m} t^2} = \\
&= e^{-i\omega^{(ad)}_{gc} t} \prod_m e^{-i\lambda_{c,m} t} \prod_m e^{-\frac{1}{2}\sigma^2_{gc;m} t^2} = \\
&= e^{-i\omega^{(ad)}_{gc} t} e^{-i\sum_m \lambda_{c,m} t} e^{-\frac{1}{2}\sum_m \sigma^2_{gc;m} t^2} = \\
&= e^{-i(\omega^{(ad)}_{gc} + \lambda_c)t - \frac{1}{2}\varsigma^2_{gc} t^2} = \\
&= e^{-i\omega_{gc} t - \frac{1}{2}\varsigma^2_{gc} t^2}
\end{aligned}
\tag{S11}
$$

where $\lambda_c = \sum_m \lambda_{c,m}$ is the total reorganization energy, $\omega_{gc} = \omega^{(ad)}_{gc} + \lambda_c$ is the vertical energy gap, and $\varsigma^2_{gc} = \sum_m \sigma^2_{gc;m}$ is the total variance, i.e., the sum of the variances along all modes.

Voigt line-shape profile

Eq. 19 in the main text gives the EGVA response function, from which the spectrum can be obtained by Fourier transformation. Since the Fourier transform of a Gaussian and a decaying exponential gives, respectively, a Gaussian and a Lorentzian function, we obtain the following expression for the linear spectrum:

$$S^{(1)}_{EGVA}(\Omega) \;\propto\; \sum_c \boldsymbol{\mu}_{gc}\boldsymbol{\mu}_{cg}\; G_{gc}(\Omega-\omega_{gc}) * L_{gc}(\Omega) \tag{S12}$$

where

$$\begin{aligned} L_{gc}(\Omega) &= \frac{2\tau_c}{(2\tau_c\Omega)^2+1} \\ G_{gc}(\Omega-\omega_{gc}) &= \exp\left[-\frac{(\Omega-\omega_{gc})^2}{2\varsigma_{gc}^2}\right] \end{aligned} \tag{S13}$$

and the symbol $*$ indicates the convolution giving rise to the *Voigt* line-shape profile.

S3 Lineshape functions second order Taylor expansion

The lineshape function $g_{ab}(t)$, involved in all expressions, reads

$$g_{ab}(t) = \frac{1}{2}\sum_m d_{a,m}d_{b,m}\left[\coth\left(\frac{\hbar\omega_m}{2k_BT}\right)(1-\cos(\omega_m t)) + i\sin(\omega_m t)\right] \tag{S14}$$

In the zero temperature limit the term $\coth\frac{\hbar\omega_m}{2k_BT} \approx 1$, so that the above expression simplifies to

$$g_{ab}(t) = \frac{1}{2}\sum_m d_{a,m}d_{b,m}\left[1-\cos(\omega_m t) + i\sin(\omega_m t)\right] \tag{S15}$$

The line-shape functions are integral transformation of the autocorrelation function describing the undamped oscillatory dynamics on the m-th normal mode harmonic potential with frequency ω_m and relative displacement with respect to the ground state equilibrium $d_{a,m}$ and $d_{b,m}$ in the a-th and b-th electronic state, respectively.[2]

S3.1 First-order response

The response function of the linear absorption involves $g_{cc}(t)$, i.e.

$$g_{cc}(t) = \frac{1}{2}\sum_m d_{c,m}^2\left[1-\cos(\omega_m t) + i\sin(\omega_m t)\right] \tag{S16}$$

The second order Taylor expansion of $g_{cc}(t)$ reads

$$\begin{aligned} g_{cc}(t) &\sim \sum_m\left[i\frac{1}{2}\omega_m d_{c,m}^2 t - \frac{1}{2}\left(\frac{1}{2}\omega_m^2 d_{c,m}^2\right)t^2\right] = \\ &= i\sum_m \lambda_{c,m}t - \frac{1}{2}\sum_m \sigma_{gc,m}^2 t^2 = \\ &= i\lambda_c t - \frac{1}{2}\varsigma_{gc}^2 t^2 \end{aligned} \tag{S17}$$

which exactly matches what we have found in the main text.

[2]In the present formulation of the line shape function, the reorganization energy $\lambda_{e,m}$ in the e-th electronic state is merged in the phase function.

S3.2 Third-order response

Let us now consider the second order Taylor expansion of the (exact) third-order response lineshape function expression for the ESA contribution, to demonstrate that this is equivalent to the one obtained in the main text in the case of adiabatic dynamics.
Under the assumption of adiabatic dynamics, the response function can be expressed as[2,3]

$$\begin{aligned} R^{(3)ESA}(t_3, t_2) = &- \sum_{e,c} \boldsymbol{\mu}_{ec}\boldsymbol{\mu}_{ce}\rho_e(t_2) e^{-i(\omega_{ec}^{(ad)} - i/2\tau_c)t_3 + \varphi_{ece}(0,t_2,t_2+t_3,0)} \\ &- \sum_{e \neq e',c} \boldsymbol{\mu}_{ec}\boldsymbol{\mu}_{ce'} e^{-i(\omega_{ee'}^{(ad)} - i/2\tau_{ee'})t_2 - i(\omega_{ec} - i/2\tau_c)t_3 + \varphi_{e'ce}(0,t_2,t_2+t_3,0)} \end{aligned} \tag{S18}$$

where the ESA contribution has been separated for *population* ($e = e'$ during t_2) and *coherence* ($e = e'$ during t_2) evolution. $\varphi_{cba}(\tau_4, \tau_3, \tau_2, \tau_1)$ are the multidimensional phase-functions encoding the time-dependent overlaps of wave-packets moving on different adiabatic surfaces. These are given by:

$$\begin{aligned} \varphi_{cba}(\tau_4, \tau_3, \tau_2, \tau_1) = &- g_{cc}(\tau_{43}) - g_{bb}(\tau_{32}) - g_{aa}(\tau_{21}) \\ &- g_{cb}(\tau_{42}) + g_{cb}(\tau_{43}) + g_{cb}(\tau_{32}) \\ &- g_{ca}(\tau_{41}) + g_{ca}(\tau_{42}) + g_{ca}(\tau_{31}) - g_{ca}(\tau_{32}) \\ &- g_{ba}(\tau_{31}) + g_{ba}(\tau_{32}) + g_{ba}(\tau_{21}) \end{aligned} \tag{S19}$$

where $\tau_{ij} = \tau_i - \tau_j$.

Applied to the *population* ESA the phase function reads

$$\begin{aligned} \varphi_{ece}(0, t_2, t_2 + t_3, 0) = &2g_{ec}(t_3) - g_{ee}(t_3) - g_{cc}(t_3) + 2\Im\left[g_{ec}(t_2) - g_{ee}(t_2) + \right. \\ &\left. -g_{ec}(t_2 + t_3) + g_{ee}(t_2 + t_3)\right] \end{aligned} \tag{S20}$$

Inserting the expression for the line-shape function $g_{ab}(t)$ one obtains

$$\varphi_{ece}(0,t_2,t_2+t_3,0) = \sum_m \Big(i\sin(\omega_m t_2)\left(d_{e,m}d_{c,m} - d_{e,m}d_{e,m}\right) - i\sin(\omega_m(t_2+t_3))\left(d_{e,m}d_{c,m} - d_{e,m}d_{e,m}\right) \\ -\frac{1}{2}\left(1-\cos(\omega_m t_3) + i\sin(\omega_m t_3)\right)\left(d_{e,m}-d_{c,m}\right)^2 \Big) \tag{S21}$$

We now apply the short time approximation with respect to t_3 in eq. S21, i.e., we consider the second order Taylor expansion of the phase function, obtaining

$$\varphi_{ece}(0,t_2,t_2+t_3,0) \sim \sum_m \Bigg(i\omega_m \left[-\cos(\omega_m t_2)\left(d_{e,m}d_{c,m} - d_{e,m}d_{e,m}\right) + \frac{1}{2}\left(d_{e,m}-d_{c,m}\right)^2 \right] t_3 \\ +\frac{1}{2} i\omega_m^2 \left[\sin(\omega_m t_2)\left(d_{e,m}d_{c,m} - d_{e,m}d_{e,m}\right) + \frac{1}{2} i \left(d_{e,m}-d_{c,m}\right)^2 \right] t_3^2 \Bigg) \tag{S22}$$

where the t_2 dependent first term of eq. S21 is cancelled by the zeroth order term in the Taylor expansion of the $t_2 + t_3$ dependent term.

At this point we can compare, order by order in t_3, the expressions for $R^{(3)ESA}(t_3, t_2)$ obtained here with those derived in the main text (eq. 40). The t_3 dependent term in eq. S22 should be compared with $-i\left(\langle \hat{H}_c\rangle_{t_2} - \langle \hat{H}_e\rangle_{t_2}\right) t_3$ (main text, eq. 35). Indeed, by expressing the reorganization energy in terms of the displacements, and by considering that in the case of adiabatic dynamics the centroid of the nuclear wave-packet evolves along a classical trajectory, for which $\langle Q_m\rangle_{e,t_2} = d_{e,m} - d_{e,m}\cos(\omega_m t_2)$, we obtain

$$-i\left(\langle \hat{H}_c\rangle_{t_2} - \langle \hat{H}_e\rangle_{t_2}\right) t_3 = \sum_m i\omega_m \left[-\frac{1}{2}\left(d_{c,m}d_{c,m} - d_{e,m}d_{e,m}\right) + \langle Q_m\rangle_{t_2}\left(d_{c,m} - d_{e,m}\right) \right] t_3 \\ = \sum_m i\omega_m \left[-\cos(\omega_m t_2)\left(d_{e,m}d_{c,m} - d_{e,m}d_{e,m}\right) + \frac{1}{2}\left(d_{e,m}-d_{c,m}\right)^2 \right] t_3 \tag{S23}$$

The t_3^2 dependent term in eq. S22, instead, can be obtained from the energy gap *pseudo*-variance $\tilde{\varsigma}_{ec}^2(t_2) = \varsigma_{ec}^2(t_2) + i2\Xi_{ec}(t_2)$. We rewrite $\varsigma_{ec}^2(t_2)$ in terms of the WP position operator variance (main text, eq. 36), and express $i2\Xi_{ec}(t_2)$ as the commutator $\langle \left[\hat{T}_m, \hat{Q}_m\right] \rangle_{t_2}$ (see

next Section), so that

$$
\begin{aligned}
-\frac{1}{2}\tilde{\varsigma}_{ec}^{2}(t_2)t_3^2 &= \sum_m -\frac{1}{2}\left[\omega_m \left\langle \left[\hat{T}_m, \hat{Q}_m\right]\right\rangle_{t_2} (d_{c,m} - d_{e,m}) + \omega_m^2 \left(d_{c,m} - d_{e,m}\right)^2 \left(\langle \hat{Q}_m^2 \rangle_{e,t_2} - \langle \hat{Q}_m \rangle_{e,t_2}^2\right)\right] t_3^2 \\
&= \sum_m \frac{1}{2} i\omega_m^2 \left[\sin\left(\omega_m t_2\right)\left(d_{e,m}d_{c,m} - d_{e,m}d_{e,m}\right) - \frac{1}{2} i \left(d_{c,m} - d_{e.m}\right)^2\right] t_3^2
\end{aligned}
\tag{S24}
$$

where we have made use of the fact that

$$
\left\langle \left[\hat{T}_m, \hat{Q}_m\right]\right\rangle_{t_2} = \left\langle \left[\hat{H}_e, \hat{Q}_m\right]\right\rangle_{t_2} = i\frac{d\,\langle \hat{Q}_m \rangle_{e,t_2}}{dt} = -i\omega_m d_{e,m} \sin\left(\omega_m t_2\right) \tag{S25}
$$

and the last step again follows from the assumed adiabatic dynamics, that gives an analytical expression for the time dependence of the WP centroid. Moreover, we also made use of the fact that the position operator variance of the adiabatically evolving wave-packet is constant, i.e.,

$$
\langle \hat{Q}_m^2 \rangle_{e,t_2} - \langle \hat{Q}_m \rangle_{e,t_2}^2 = 1/2 \tag{S26}
$$

An analytical expression for the linear chirp $\Xi_{ec}(t_2)$ term is thus obtained, in the assumption that the system always follows an adiabatic dynamics

$$
\begin{aligned}
\Xi_{ec}(t_2) &= \frac{1}{2}\omega_m^2 d_{e,m} \sin\left(\omega_m t_2\right)\left(d_{c,m} - d_{e,m}\right) = \\
&= \frac{1}{2}\omega_m^2 \left\langle P_m \right\rangle_{e,t_2} \left(d_{c,m} - d_{e,m}\right)
\end{aligned}
\tag{S27}
$$

where $\langle P_m \rangle_{e,t_2} = d_{e,m}\sin(\omega t_2)$ is the expectation value of the momentum operator in dimensionless coordinates.

S4 The commutator $\left[\hat{H}_e, \hat{H}_c\right]$

Let us first rewrite $\hat{H}_{c,m}$ as $\hat{H}_{c,m} = \hat{H}_{e,m} + \frac{1}{2}\omega_m \left(d_{e,m} - d_{c,m}\right)^2 - \omega_m d_{e,m} \left(d_{e,m} - d_{c,m}\right) + \omega_m \hat{Q}_m \left(d_{e,m} - d_{c,m}\right)$, so that $\left[\hat{H}_{e,m}, \hat{H}_{c,m}\right]$ simplifies to $\omega_m \left(d_{e,m} - d_{c,m}\right) \left[\hat{T}_m, \hat{Q}_m\right]$. Therefore, for the population contribution, we have

$$\begin{aligned}
\langle\chi_{e,m}(t_2)| \left[\hat{H}_{e,m}, \hat{H}_{c,m}\right] |\chi_{e,m}(t_2)\rangle &= \sum_m \omega_m(d_{e,m} - d_{c,m}) \langle\chi_{e,m}(t_2)| \left[\hat{T}_m, \hat{Q}_m\right] |\chi_{e,m}(t_2)\rangle = \\
&= \sum_m \omega_m(d_{e,m} - d_{c,m}) \langle\chi_{e,m}(t_2)| \left[-\frac{\omega_m}{2}\frac{\partial^2}{\partial Q_m^2}, \hat{Q}_m\right] |\chi_{e,m}(t_2)\rangle = \\
&= -\sum_m \omega_m^2(d_{e,m} - d_{c,m}) \langle\chi_{e,m}(t_2)|\frac{\partial}{\partial Q_m}|\chi_{e,m}(t_2)\rangle = \\
&= -i\sum_m \omega_m^2(d_{e,m} - d_{c,m}) \langle\hat{P}_m\rangle_{e,t_2}
\end{aligned} \tag{S28}$$

where $\langle\hat{P}_m\rangle_{t_2}$ is the expectation value of the momentum operator.

S5 $R^{(3)ESA}_{WPO^*}(t_3, t_2)$ ***coherence*** term in the EGVA approach

In what follows we consider the *coherence* term of the $R^{(3)ESA}_{WPO^*}(t_3, t_2)$, labled here as $R^{(3)ESA,coh}_{WPO^*}(t_3, t_2)$, aimed at deriving the corresponding EGVA expression. First, we start from eq. 28 of the main text, which gives

$$R^{(3)ESA,coh}_{WPO^*}(t_3, t_2) = -\mathcal{N}_\mu \sum_{e\neq e',c} \sqrt{\rho_e(t_2)\rho_{e'}(t_2)}\boldsymbol{\mu}_{ec}\boldsymbol{\mu}_{ce'} e^{-i\omega^{(ad)}_{ec}t_3} \langle\bar{\chi}^\mu_e(t_2)| e^{i\hat{H}_e t_3} e^{-i\hat{H}_c t_3} |\bar{\chi}^\mu_{e'}(t_2)\rangle e^{-t_3/2\tau_c} \tag{S29}$$

A significant difference with respect to the $R^{(3)ESA,pop}_{WPO^*}(t_3, t_2)$ term, is that the overlap between the bra and ket (normalized) wave-packet is not equal to 1. In fact

$$\langle\bar{\chi}^\mu_e(t_2)|\bar{\chi}^\mu_{e'}(t_2)\rangle = \begin{cases} 1 & \text{if } e = e' \\ \langle 1\rangle_{t_2} \in \mathbb{C} & \text{if } e \neq e' \end{cases} \tag{S30}$$

where $\langle 1\rangle_{t_2}$ is a generic t_2 function which can assume whatever value between -1 and 1.

Let us focus on the t_3 wave-packet overlap $\langle\bar{\chi}^\mu_e(t_2)| e^{i\hat{H}_e t_3} e^{-i\hat{H}_c t_3} |\bar{\chi}^\mu_{e'}(t_2)\rangle$. Following similar steps to those shown in the main text for the corresponding *population* term, we get

$$\begin{aligned} e^{i\hat{H}_e t_3} &= e^{i\frac{\langle\hat{H}_e\rangle_{t2}}{\langle 1\rangle_{t2}}t_3} e^{i\left(\hat{H}_e - \frac{\langle\hat{H}_e\rangle_{t2}}{\langle 1\rangle_{t2}}\right)t_3} \\ e^{-i\hat{H}_c t_3} &= e^{-i(\hat{H}_c - \frac{\langle\hat{H}_c\rangle_{t2}}{\langle 1\rangle_{t2}})t_3} e^{-i\frac{\langle\hat{H}_c\rangle_{t2}}{\langle 1\rangle_{t2}}t_3} \end{aligned} \tag{S31}$$

where we have introduced the matrix element $\langle\hat{H}_a\rangle_{t_2} = \langle\bar{\chi}^\mu_e(t_2)| \hat{H}_a |\bar{\chi}^\mu_{e'}(t_2)\rangle$ (with $a \in \mathcal{E}, \mathcal{C}$) weighted by the time dependent overlap $\langle 1\rangle_{t_2} = \langle\bar{\chi}^\mu_e(t_2)|\bar{\chi}^\mu_{e'}(t_2)\rangle$. The need of this weighting factor will become apparent later.[3]

[3]Note that $\langle 1\rangle_{t_2}$ can in principle be also equal to zero. This can happen when: at least one of the two electronic states e and e' is not populated; the bra and ket WP reside in completely different regions of the coordinate space, and the overlap is therefore null. In both cases one can safely consider the full S29 term to be null, and thus avoiding considering the derivation that follows. Thus, for such derivation, we assume $\langle 1\rangle_{t_2} \neq 0$.

We thus rewrite the wave function overlap of eq. S29 as:

$$e^{-i\frac{(\langle \hat{H}_c\rangle_{t2}-\langle \hat{H}_e\rangle_{t2})}{\langle 1\rangle_{t2}}t_3}\langle e^{i\left(\hat{H}_e-\frac{\langle \hat{H}_e\rangle_{t2}}{\langle 1\rangle_{t2}}\right)t_3}e^{-i\left(\hat{H}_c-\frac{\langle \hat{H}_c\rangle_{t2}}{\langle 1\rangle_{t2}}\right)t_3}\rangle_{t_2} \tag{S32}$$

where we have have adopted the same notation of the main paper in which $\langle\cdots\rangle$ is used to highlight the matrix element: $\langle \bar{\chi}_e^{\mu}(t_2)|\cdots|\bar{\chi}_{e'}^{\mu}(t_2)\rangle$. When the maximum value assumed by t_3 is very small (*short time approximation*) the propagator in the last equation is well approximated by the it's second order Taylor expansion in t_3. This leads to:

$$\begin{aligned}
&\left\langle\left[1+i\left(\hat{H}_e-\frac{\langle\hat{H}_e\rangle_{t_2}}{\langle 1\rangle_{t_2}}\right)t_3-\frac{1}{2}\left(\hat{H}_e-\frac{\langle\hat{H}_e\rangle_{t_2}}{\langle 1\rangle_{t_2}}\right)^2 t_3^2\right]\times\right.\\
&\left.\times\left[1-i\left(\hat{H}_c-\frac{\langle\hat{H}_c\rangle_{t_2}}{\langle 1\rangle_{t_2}}\right)t_3-\frac{1}{2}\left(\hat{H}_c-\frac{\langle\hat{H}_c\rangle_{t_2}}{\langle 1\rangle_{t_2}}\right)^2 t_3^2\right]\right\rangle=\\
&=\langle 1\rangle_{t_2}-\frac{1}{2}\left[\langle\left(\hat{H}_e-\frac{\langle\hat{H}_e\rangle_{t_2}}{\langle 1\rangle_{t_2}}\right)^2\rangle+\langle\left(\hat{H}_c-\frac{\langle\hat{H}_c\rangle_{t_2}}{\langle 1\rangle_{t_2}}\right)^2\rangle-\right.\\
&\left.-2\langle\left(\hat{H}_e-\frac{\langle\hat{H}_e\rangle_{t_2}}{\langle 1\rangle_{t_2}}\right)\left(\hat{H}_c-\frac{\langle\hat{H}_c\rangle_{t_2}}{\langle 1\rangle_{t_2}}\right)\rangle\right]t_3^2=\\
&=\langle 1\rangle_{t_2}-\frac{1}{2}\langle\left[\left(\hat{H}_e-\hat{H}_c\right)-\left(\frac{\langle\hat{H}_e\rangle_{t_2}-\langle\hat{H}_c\rangle_{t_2}}{\langle 1\rangle_{t_2}}\right)\right]^2\rangle t_3^2-\frac{1}{2}\langle\left[\hat{H}_e,\hat{H}_c\right]\rangle t_3^2
\end{aligned} \tag{S33}$$

where first order term in t_3 have been canceled thanks to the $\langle 1\rangle_{t_2}$ weighting factor. At this point, one would be tempted to express the the t_3^2 dependent terms in analogy to the expression derived for the *population* contribution of the response function. To do so, we define the following quantities:

$$\sigma^2_{ee'c,m}(t_2)=\langle\left[\left(\hat{H}_e-\hat{H}_c\right)-\left(\frac{\langle\hat{H}_e\rangle_{t_2}-\langle\hat{H}_c\rangle_{t_2}}{\langle 1\rangle_{t_2}}\right)\right]^2\rangle \tag{S34}$$

where the subscript $ee'c$ denotes the fact that this term is computed on the ee' coherence, and to the final state accessed via the interaction with the probe pulse is c. $\sigma^2_{ee'c,m}(t_2)$ is the

equivalent of the variance defined for the ESA population term, and we similarly define the equivalent of the pseudovariance as

$$\tilde{\sigma}^2_{ee'c,m}(t_2) = \sigma^2_{ee'c,m}(t_2) + \langle \left[\hat{H}_{e,m}, \hat{H}_{c,m}\right] \rangle \tag{S35}$$

At this point, by noticing that:

$$\langle 1 \rangle_{t_2} \left(1 - \frac{\tilde{\sigma}^2_{ee'c,m}(t_2)}{2\langle 1 \rangle_{t_2}} t_3^2\right) \sim \langle 1 \rangle_{t_2} e^{-\frac{\tilde{\sigma}^2_{ee'c,m}(t_2)}{2\langle 1 \rangle_{t_2}} t_3^2} \tag{S36}$$

the overlap of equation S29 can be finally rewritten as:

$$\langle \bar{\chi}^{\mu}_{e}(t_2) | \, e^{i\hat{H}_e t_3} e^{-i\hat{H}_c t_3} \, | \bar{\chi}^{\mu}_{e'}(t_2) \rangle = \langle 1 \rangle_{t_2} e^{-i\frac{\langle \hat{H}_c \rangle_{t2} - \langle \hat{H}_e \rangle_{t2}}{\langle 1 \rangle_{t2}} t_3 - \frac{\tilde{\sigma}^2_{ee'c,m}(t_2)}{2\langle 1 \rangle_{t_2}} t_3^2} \tag{S37}$$

which is reminescent of the similar expression obtained for the ESA population term, even if much more involved. Interestingly, if the WP overlap approaches zero, the signal converges to zero. This highlights the fact that the ESA signal of the *coherence* can contribute to the total spectra only when a significant overlap between the e and e' WP is observed.

S6 WPO* vs EGVA LA: high and low frequency modes

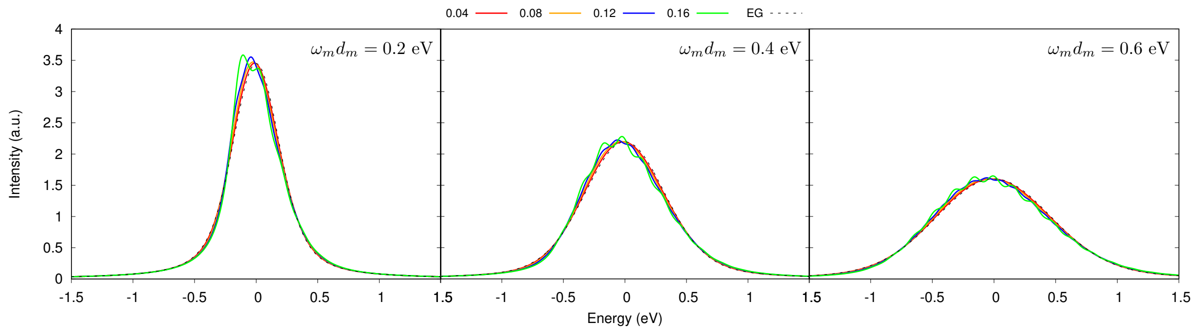

Figure s8: Comparison of the EGVA (dashed black line) and analytical LA spectra for a simple model system with the excited state c coupled to a single vibrational mode of frequency ω_m and displacement d_m. ω_m ranges between 0.04 eV and 0.16 eV. The excited state lifetime is set to $\tau_c = 3$ fs. Note how the EGVA approximation improves for lower mode frequencies. Note also that at larger displacements, a quasi-symmetric shape of the spectrum is recovered, which improves the quality of the EGVA approximation also for higher mode frequencies.

In order to assess the quality of the EGVA approximation for high- and low-frequency modes, we built a simple model system with two dipole-coupled electronic states, g and c, with the latter coupled to a single mode, m, with frequency ω_m and displacement (with respect to the ground state minimum) d_m. The mode frequency was varied between 0.04 and 0.16 eV, which correspond to a mode period of ca. 103 and 26 fs, respectively.[4] We show spectra with both $\tau_c = 3$ and $\tau_c = 5$ fs.

The rationale behind this analysis is the following: since the EGVA approximation is based on the time scale separation between core-excited state lifetime (supposed short) and period of the vibrations (supposed longer), the lower the mode frequency, the better the quality of the EGVA approximation should be. Indeed, within the $g - c$ coherence lifetime (whose Fourier transform gives rise to the -linear- spectrum) the system does not present any significant motion of the WP along the low-frequency modes (which might not be strictly true for high frequency modes).

[4]Note that in generic molecular systems the highest frequency modes are $C - H$, $N - H$ and $O - H$ stretching, with frequencies of about 3000 cm^{-1}, i.e., ~0.4 eV. We choose not to consider such extremely high frequencies here as these modes do not typically affect the spectra (i.e., the various electronic state PESs are not displaced along these modes).

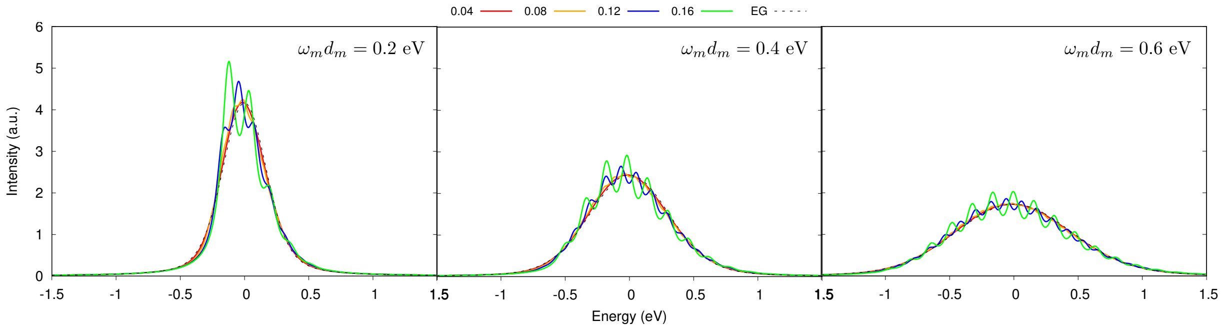

Figure s9: Comparison of the EG (dashed black line) and analytical LA spectra for a simple model system with the excited state c coupled to a single vibrational mode of frequency ω_m and displacement d_m. The excited state lifetime is set to $\tau_c = 5$ fs. Note how the EG approximation improves for lower mode frequencies. Note also that at larger displacements, a quasi-symmetric shape of the spectrum is recovered, which improves the quality of the EG approximation also for higher mode frequencies. The frequency values are reported in eV.

The results are shown in Figure s8 and s9 for three gradient values, $\omega_m d_m$. Note that, for a given gradient value, the EGVA spectrum is independent on the mode frequency. As expected, for $\omega_m = 0.04$ and 0.08 eV, the EGVA linear spectrum is on top of the exact one; $\omega_m = 0.12$ eV (i.e., a period of 34 fs) is nicely reproduced, while significant deviations in the spectral shape are observed for $\omega_m = 0.16$ eV. In particular, the EGVA approximation in unable to reproduce the asymmetry of the spectral shape and the vibronic peaks. Interestingly, for large displacements, whereby a large number of high-energy vibrational states are active, the band-shape symmetry is recovered, and the quality of the EGVA spectra improves at higher mode frequencies. A similar analysis is reported in Figure s9, employing a lifetime $\tau_c = 5$ fs. The differences between the EGVA and the exact spectra are indeed more prominent.

S7 WPO* vs EGVA LA at various values of τ_c

Figure s10 shows the comparison of the XAS spectrum from the 11 brightest $g \to \mathcal{C}$ transitions, for various values of τ_c: 3, 5 and 7 fs. In the main text we presented results that always employ $\tau_c = 3$ fs. It is clear that $\tau_c = 7$ fs represent a limit of extremely high vibronic resolution: since we are not explicitly accounting for static disorder, choosing such a large τ_c value gives spectra that are unrealistically resolved.

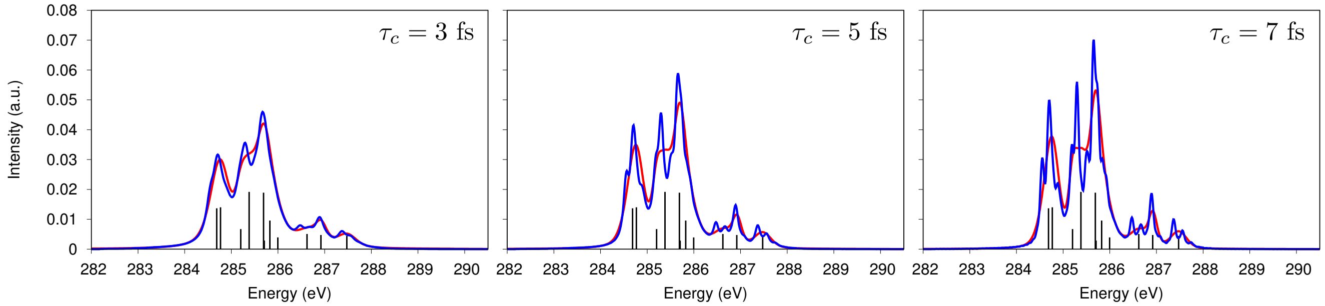

Figure s10: Comparison of WPO* (blue curve) and EGVA (red curve) XAS spectra setting τ_c to a) 3 fs, b) 5 fs and c) 7 fs.

S8 EGVA XAS spectrum: variable vs constant variance

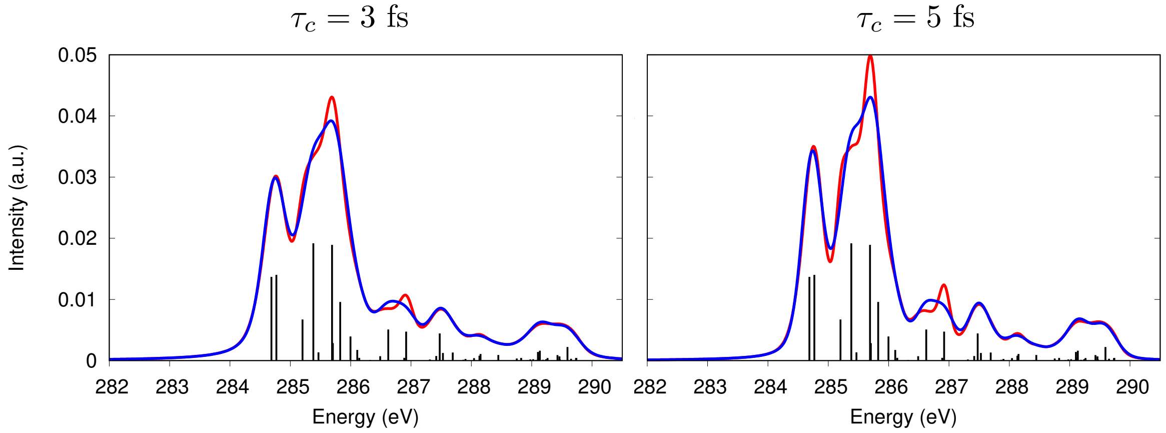

Figure s11: Comparison of total XAS spectra obtained at the EGVA level of theory, employing: the calculated ς_{gc}^2 value (red curve), and a constant ς_{gc}^2 value, set to the average of the variances values, i.e., to 0.019 eV^2. Note that the latter tends to smooth out some spectral features. $\tau_c = 3$ fs (*left*) and 5 fs (*right*).

S9 Energy gaps, energy-gap variances and TDMs

Table S1: Energy gap (eV), Energy gap variance (eV^2) and TDMs for the 75 $g \rightarrow c$ transitions that builds the total pyrene XAS spectrum. First part.

**State**	EG (eV)	EGV (eV^2)	TDM^2 (a.u.)
c1	284.69	0.026	3.9×10^{-03}
c2	284.77	0.013	4.0×10^{-03}
c3	285.15	0.029	2.3×10^{-13}
c4	285.20	0.004	1.9×10^{-03}
c5	285.32	0.017	1.2×10^{-14}
c6	285.38	0.017	5.5×10^{-03}
c7	285.47	0.011	3.8×10^{-04}
c8	285.69	0.008	5.4×10^{-03}
c9	285.71	0.034	8.2×10^{-04}
c10	285.83	0.018	2.7×10^{-03}
c11	286.00	0.030	1.1×10^{-03}
c12	286.11	0.006	5.0×10^{-04}
c13	286.14	0.035	1.3×10^{-04}
c14	286.32	0.012	3.2×10^{-05}
c15	286.49	0.008	2.1×10^{-04}
c16	286.62	0.030	1.5×10^{-03}
c17	286.89	0.031	1.3×10^{-04}
c18	286.92	0.004	1.4×10^{-03}
c19	286.95	0.009	4.3×10^{-16}
c20	286.95	0.013	3.0×10^{-15}
c21	287.31	0.017	5.0×10^{-05}
c22	287.42	0.013	2.1×10^{-04}
c23	287.48	0.022	1.3×10^{-03}
c24	287.53	0.018	3.6×10^{-04}
c25	287.69	0.014	3.8×10^{-04}
c26	287.74	0.043	3.8×10^{-07}
c27	287.87	0.018	3.9×10^{-05}
c28	287.90	0.014	7.8×10^{-05}
c29	288.04	0.030	1.1×10^{-04}
c30	288.06	0.020	3.5×10^{-13}

Table S2: Energy gap (eV), Energy gap variance (eV^2) and TDMs for the 75 $g \rightarrow c$ transitions that builds the total pyrene XAS spectrum. Second part.

**State**	EG (eV)	EGV (eV^2)	TDM^2 (a.u.)
c31	288.12	0.007	3.9×10^{-06}
c32	288.13	0.026	4.1×10^{-13}
c33	288.13	0.011	2.3×10^{-04}
c34	288.15	0.029	5.4×10^{-05}
c35	288.15	0.010	3.1×10^{-04}
c36	288.18	0.039	1.1×10^{-13}
c37	288.40	0.027	3.0×10^{-16}
c38	288.42	0.026	1.6×10^{-05}
c39	288.45	0.013	2.6×10^{-04}
c40	288.58	0.021	1.8×10^{-08}
c41	288.71	0.025	4.6×10^{-06}
c42	288.74	0.010	1.2×10^{-05}
c43	288.75	0.012	9.7×10^{-05}
c44	288.78	0.010	4.8×10^{-16}
c45	288.78	0.054	1.3×10^{-05}
c46	288.82	0.026	1.3×10^{-04}
c47	288.84	0.019	9.1×10^{-06}
c48	288.91	0.015	2.0×10^{-15}
c49	288.93	0.042	2.1×10^{-06}
c50	288.93	0.018	2.3×10^{-05}
c51	288.95	0.007	2.1×10^{-06}
c52	288.98	0.014	5.4×10^{-05}
c53	289.02	0.018	6.5×10^{-05}
c54	289.08	0.032	3.4×10^{-05}
c55	289.10	0.022	4.0×10^{-04}
c56	289.13	0.017	1.3×10^{-04}
c57	289.14	0.026	4.6×10^{-04}
c58	289.19	0.016	4.0×10^{-05}
c59	289.23	0.015	3.5×10^{-05}
c60	289.24	0.054	6.6×10^{-05}

Table S3: Energy gap (eV), Energy gap variance (eV^2) and TDMs for the 75 $g \to c$ transitions that builds the total pyrene XAS spectrum. Third part.

**State**	EG (eV)	EGV (eV^2)	TDM^2 (a.u.)
c61	289.26	0.012	1.2×10^{-04}
c62	289.27	0.031	7.6×10^{-09}
c63	289.29	0.006	6.3×10^{-13}
c64	289.32	0.010	7.2×10^{-13}
c65	289.34	0.025	6.0×10^{-07}
c66	289.40	0.018	2.6×10^{-06}
c67	289.42	0.012	2.3×10^{-13}
c68	289.43	0.010	2.6×10^{-04}
c69	289.46	0.023	2.0×10^{-04}
c70	289.47	0.014	5.7×10^{-05}
c71	289.54	0.006	2.5×10^{-13}
c72	289.59	0.016	6.4×10^{-04}
c73	289.65	0.011	8.7×10^{-05}
c74	289.73	0.018	1.2×10^{-04}
c75	289.74	0.005	1.3×10^{-04}

Table S4: Energy gap (eV), Energy gap variance (eV^2) (at $t_2 = 0$ fs) and TDMs for the 28 $\mathcal{G}/\mathcal{E} \rightarrow \mathcal{C}$ transitions that builds the total pyrene TRXAS spectrum (11 dipole coupled to g, 12 dipole coupled to S_1, and 6 dipole coupled to S_2). Note that the EGV for the $S_1 \rightarrow \mathcal{C}$ transitions is not reported as, at time 0, the WP does not reside on the S_1 state (yet).

**In. State**	**Fin. State**	EG (eV)	EGV (eV^2)	TDM^2 (a.u.)
g	c_1	284.69	0.026	3.9×10^{-03}
g	c_2	284.77	0.013	4.0×10^{-03}
g	c_4	285.20	0.004	1.9×10^{-03}
g	c_6	285.38	0.017	5.5×10^{-03}
g	c_8	285.69	0.008	5.4×10^{-03}
g	c_9	285.71	0.034	8.2×10^{-04}
g	c_{10}	285.83	0.018	2.7×10^{-03}
g	c_{11}	286.00	0.030	1.1×10^{-03}
g	c_{16}	286.62	0.030	1.5×10^{-03}
g	c_{18}	286.92	0.004	1.4×10^{-03}
g	c_{23}	287.48	0.022	1.3×10^{-03}
S_1	c_3	281.92	-	1.02×10^{-03}
S_1	c_7	282.23	-	1.27×10^{-03}
S_1	c_{28}	284.67	-	1.45×10^{-03}
S_1	c_{29}	284.81	-	9.69×10^{-04}
S_1	c_{30}	284.83	-	2.03×10^{-03}
S_1	c_{38}	285.19	-	1.34×10^{-03}
S_1	c_{48}	285.68	-	1.28×10^{-03}
S_1	c_{52}	285.75	-	1.21×10^{-03}
S_1	c_{53}	285.79	-	1.62×10^{-03}
S_1	c_{63}	286.06	-	1.27×10^{-03}
S_1	c_{64}	286.09	-	2.25×10^{-03}
S_1	c_{65}	286.10	-	1.06×10^{-03}
S_2	c_1	280.94	0.002	5.67×10^{-04}
S_2	c_{22}	283.67	0.001	1.03×10^{-03}
S_2	c_{24}	283.78	0.006	7.24×10^{-04}
S_2	c_{49}	285.18	0.012	1.06×10^{-03}
S_2	c_{50}	285.18	0.019	1.02×10^{-03}
S_2	c_{58}	285.44	0.007	1.95×10^{-03}

S10 Pyrene LA: full mode models vs reduced (15) modes model

Figure s12 shows the comparison between the $g \to c_1$ transition for the full modes pyrene model comprising 49 normal modes, in red, and the reduced pyrene model comprising 15 normal modes, in blue. The 15 modes were selected to describe the nonadiabatic dynamics that occur in the $\mathcal{E}$ manifold. Interestingly, it turned out that the $g \to c_1$ is strongly coupled along some of the left out modes. A strategy to account for the left out modes via analytical line-shape functions (while still accounting for the 15 selected modes via the WPO approach) is described in Ref. 4.

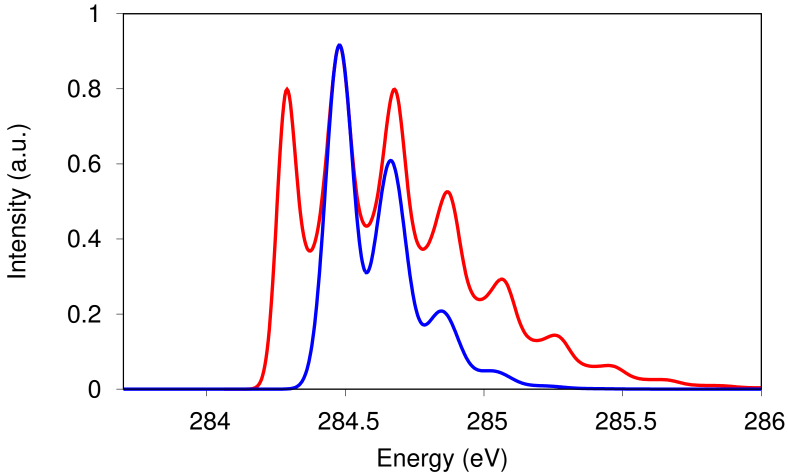

Figure s12: Comparison of the $g \to c_1$ linear absorption spectrum for the 49 modes pyrene model (red curve) and the 15 modes pyrene model (blue curve). The linear absorption spectra has been normalized to a common value.

S11 Response function Fourier transform: technical details

The valence-to-core transitions cover a range of a few eV, say around 10 eV. This is problematic when performing the FT. In fact, we have a discrete time step of 0.5 fs, that is going to produce aliasing of the spectra every $\sim$10 eV. But now we are precisely in this situation (one does not encounter such a problem in the UV/Vis window, as in that case the spectrum is typically only a few eV wide). This requires special attention when performing the FT.

One might reduce the time-step of the quantum dynamics, at the same time increasing the computational cost of such calculations. We have solved the problem in a different (and more efficient) way: every transition has its own central frequency. We shift this to 2.5 eV. Then we make the FT in between 0 and 5 eV (i.e. around the center of the transition, assuming that 5 eV is a large enough range to cover the full bandwidth of the given transition). After completing the FT, we recenter and add this chunk of spectrum to the total spectrum in the right place, i.e. shifting the center from 2.5 eV to the actual central frequency. Since everything is done in discrete grids, one should take care of the alignment of the grids for different transitions.

References

(1) Segatta, F.; Ruiz, D. A.; Aleotti, F.; Yaghoubi, M.; Mukamel, S.; Garavelli, M.; Santoro, F.; Nenov, A. Nonlinear Molecular Electronic Spectroscopy via MCTDH Quantum Dynamics: From Exact to Approximate Expressions. *Journal of Chemical Theory and Computation* **2023**,

(2) Mukamel, S. *Principles of Nonlinear Optical Spectroscopy*; Oxford University Press: New York, 1995.

(3) Abramavicius, D.; Palmieri, B.; Voronine, D. V.; Šanda, F.; Mukamel, S. Coherent Multidimensional Optical Spectroscopy of Excitons in Molecular Aggregates Quasiparticle versus Supermolecule Perspectives. *Chemical Reviews* **2009**, *109*, 2350–2408.

(4) Montorsi, F.; Aranda, D.; Garavelli, M.; Santoro, F.; Segatta, F. Spectroscopy from quantum dynamics: a mixed wave function/analytical line shape functions approach. *Theoretical Chemistry Accounts* **2023**, *142*.
